# Structural Diversity in Alkali Metal and Alkali Metal Magnesiate Chemistry of the Bulky 2,6‐Diisopropyl‐*N*‐(trimethylsilyl)anilino Ligand

**DOI:** 10.1002/chem.201602683

**Published:** 2016-08-30

**Authors:** M. Ángeles Fuentes, Andoni Zabala, Alan R. Kennedy, Robert E. Mulvey

**Affiliations:** ^1^ WestCHEMDepartment of Pure and Applied ChemistryUniversity of StrathclydeGlasgowG1 1XLUK

**Keywords:** alkali metals, amides, bases, magnesiates, metalation, structure elucidation

## Abstract

Bulky amido ligands are precious in s‐block chemistry, since they can implant complementary strong basic and weak nucleophilic properties within compounds. Recent work has shown the pivotal importance of the base structure with enhancement of basicity and extraordinary regioselectivities possible for cyclic alkali metal magnesiates containing mixed *n*‐butyl/amido ligand sets. This work advances alkali metal and alkali metal magnesiate chemistry of the bulky arylsilyl amido ligand [N(SiMe_3_)(Dipp)]^−^ (Dipp=2,6‐*i*Pr_2_‐C_6_H_3_). Infinite chain structures of the parent sodium and potassium amides are disclosed, adding to the few known crystallographically characterised unsolvated s‐block metal amides. Solvation by *N*,*N*,*N′*,*N′′*,*N′′*‐pentamethyldiethylenetriamine (PMDETA) or *N*,*N*,*N′*,*N′*‐tetramethylethylenediamine (TMEDA) gives molecular variants of the lithium and sodium amides; whereas for potassium, PMDETA gives a molecular structure, TMEDA affords a novel, hemi‐solvated infinite chain. Crystal structures of the first magnesiate examples of this amide in [MMg{N(SiMe_3_)(Dipp)}_2_(μ‐*n*Bu)]_∞_ (M=Na or K) are also revealed, though these breakdown to their homometallic components in donor solvents as revealed through NMR and DOSY studies.

## Introduction

Alkali metal amides of bulky secondary amines, [(MNR_2_)_*n*_], are popular tools in many synthetic campaigns due to a complementary combination of strong deprotonating power and weak nucleophilicity. Most common are lithium 1,1,1,3,3,3‐hexamethyldisilazide (LiHMDS), lithium diisopropylamide (LiDA) and lithium 2,2,6,6‐tetramethylpiperidide (LiTMP), referred to collectively as the “utility amides”.^1^ Though all these amides are useful in their monometallic form, the performance of the TMP anion in C−H deprotonation applications can be significantly enhanced by incorporating it within multicomponent systems such as the turbo‐Hauser base [TMPMgCl⋅LiCl]^2^ or magnesiate [(TMEDA)Na(μ‐TMP)(μ‐*n*Bu)Mg(TMP)]^3^ (TMEDA=*N*,*N*,*N′*,*N′*‐tetramethylethylenediamine) or alternatively by administering LiTMP in combination with an organometallic [e.g., Al(*i*Bu)_3_,^4^ (TMP)Al(*i*Bu)_2_,^5^ Zn(TMP)_2_ 6] or salt (MgCl_2_, ZnCl_2_ or CuCN)^7^ trapping agent, which can drive equilibria to products by stabilising sensitive intermediates through lower polarity M−C bonds (M=Al, Mg, Zn). Curiosity in these new TMP reagent mixtures is increased by the fact their chemistry can be considered cooperative in origin, implemented through distinct components working together through either synchronised or stepwise mechanisms. A leading example of the former is hexanuclear sodium magnesiate [Na_4_Mg_2_(TMP)_6_(*n*Bu)_2_],^8^ a bimetallic ring complex with two pendant butyl base units, in functioning as a template to effect *ortho*–*meta′* or *meta*–*meta′* dideprotonation of a suite of aromatic substrates, superseding directed *ortho*‐metallation (D*o*M),^9^ in reactions irreproducible by the separated monometallic components.

**Figure 1:**
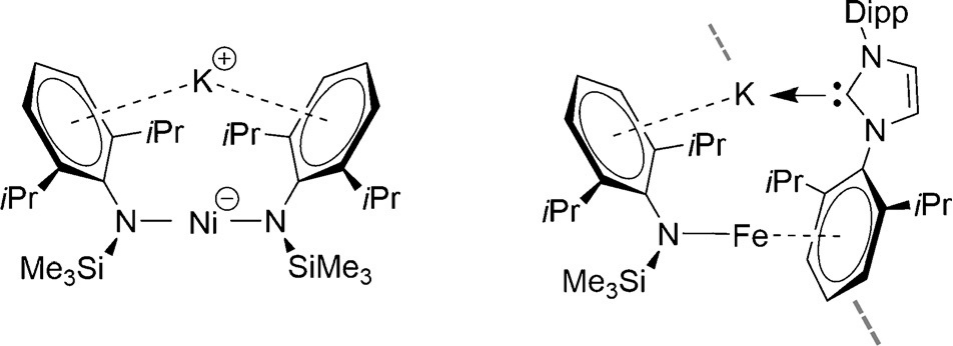
Low‐coordinate Ni and Fe complexes showing interaction of potassium with π‐system as reported by Tilley.^14^

Casting the net wider for other bulky amides that might possess interesting cooperativity in magnesiate modifications our attention was drawn to the arylsilyl amido ligand [N(SiMe_3_)(Dipp)]^−^ (where Dipp=2,6‐diisopropylphenyl=2,6‐*i*Pr_2_‐C_6_H_3_). Its metallo chemistry is well developed, with Power^10^ recently reporting the extraordinary finding that C−H⋅⋅⋅H−C dispersion forces between two such eclipsed amido ligands helped stabilise linear, two‐coordinate Fe, Co and Ni bis(amides), while Tilley^11^ demonstrated the catalytic capability of the Ni bis(amide) in carbon–carbon cross‐coupling reactions. Though [(Li{N(SiMe_3_)(Dipp)})_2_] commonly acts as a transfer agent to generate such transition‐metal amides, surprisingly the alkali metal chemistry of this amide has many gaps. While Roesky^12^ has determined the dimeric structure of [(Li{N(SiMe_3_)(Dipp)})_2_] and solvated structures such as Anwander's [Li{N(SiMe_3_)(Dipp)}(THF)_3_]^13^ are known, the sodium and potassium congeners have not been crystallographically characterised. However, potassium has been observed in interesting transition‐metal bimetallic systems reported by Tilley^14^ that demonstrate the large, soft alkali metal's propensity for engaging with arene‐π systems (Figure 1).^15^ While Ruhlandt‐Senge^16^ has described two‐coordinate [Mg{N(SiMe_3_)(Dipp)}_2_] and the THF solvate [(THF)_2_Mg{N(SiMe_3_)(Dipp)}(*n*Bu)], and [Mg{N(SiMe_3_)(Dipp)}_2_(OEt_2_)]^17^ was described by our group, to the best of our knowledge no alkali metal magnesiate has hitherto been reported. Therefore this study sets out to fill in important gaps in the alkali metal chemistry of [N(SiMe_3_)(Dipp)]^−^ and to synthesise and characterise the first magnesiate examples as a prerequisite to determining whether this amide also possesses synergistic template base potential.1

## Result and Discussion

### Synthetic studies

A total of ten new crystalline compounds were prepared in this study. These comprise the solvent‐free parent sodium and potassium amides [Na{N(SiMe_3_)(Dipp)}]_∞_ (**1**) and [K{N(SiMe_3_)(Dipp)}]_∞_ (**2**); the PMDETA (*N*,*N*,*N′*,*N′′*,*N′′*‐pentamethyldiethylenetriamine) solvates [Li{N(SiMe_3_)(Dipp)}(PMDETA)] (**3**), [Na{N(SiMe_3_)(Dipp)}(PMDETA)] (**4**) and [{K{N(SiMe_3_)(Dipp)}(PMDETA)}_2_] (**5**); the TMEDA (*N*,*N*,*N′*,*N′*‐tetramethylethylenediamine) solvates [Li{N(SiMe_3_)(Dipp)}(TMEDA)] (**6**), [{Na{N(SiMe_3_)(Dipp)}(TMEDA)}_2_] (**7**) and [{K{N(SiMe_3_)(Dipp)}}_2_(TMEDA)]_∞_ (**8**); and the heavier alkali metal magnesiates [NaMg{N(SiMe_3_)(Dipp)}_2_(μ‐*n*Bu)]_∞_ (**9**) and [KMg{N(SiMe_3_)(Dipp)}_2_(μ‐*n*Bu)]_∞_ (**10**). Polyamines PMDETA and TMEDA are popular chelating and solubility‐enhancing ligands in alkali metal chemistry;^18^ they generally help to deaggregate the parent unsolvated compounds as reflected here, though interestingly deaggregation has not occurred in the hemi‐TMEDA solvate **8**.^19^

**Scheme 1:**
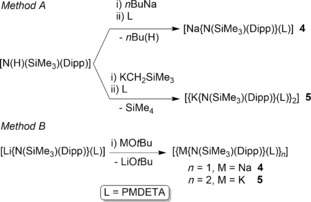
Alternative methods to prepare complexes **4** and **5**.

Monometallic complexes **1**–**8** were all synthesised by deprotometallation of the starting amine N(H)(SiMe_3_)(Dipp) by a metal alkyl reagent. In the lithium and sodium cases this was the relevant metal *n*‐butyl reagent, whereas the lower stability of potassium alkyls necessitated switching to the more stable silylalkyl reagent KCH_2_SiMe_3_, which unlike the *n*‐butyl reagent does not possess any β‐hydrogen atoms, and so avoids possible decomposition by such an elimination reaction. Ruhlandt‐Senge^16^ previously made [K{N(SiMe_3_)(Dipp)}] (probably as a THF solvate) by deprotonation of the amine with potassium hydride in THF and used it in situ to generate Group 2 bis(amides) by salt metathesis with the relevant Group 2 metal iodide. Anwander^13^ used a similar salt metathesis approach to make a series of rare earth metal amide complexes, during which he isolated [K{N(SiMe_3_)(Dipp)}] in powder form and crystallised the tris(THF) solvate of [Li{N(SiMe_3_)(Dipp)}] and determined its monomeric structure. In our study we observed no benefit in making the PMDETA solvates **4** and **5** through the salt metathesis of the lithium amide [Li{N(SiMe_3_)(Dipp)}] with sodium *tert*‐butoxide and potassium *tert*‐butoxide, respectively, as crystalline yields of all the products were about 50 % from both deprotometallation and metathesis methods (Scheme 1). It is worth noting that irrespective of the method employed, ^1^H NMR monitoring of the filtrates from reaction solutions showed essentially quantitative conversions of the amine to amide in all cases. Both the starting metal reagent and, where relevant, the donor solvent employed in these reactions were added in slight excess compared to the parent amine.1

**Scheme 2:**
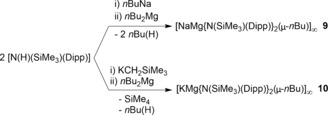
Method to prepare alkali metal magnesiates **9** and **10**.

Deprotometallation was used also in the syntheses of magnesiates **9** and **10** (Scheme 2). *n*‐Butylsodium and trimethylsilylmethylpotassium were utilised as above to generate the respective heavier alkali metal amide from the amine, which in turn was reacted in a co‐complexation approach with the *n*‐butylmagnesium amide to afford the monoalkyl–bisamidomagnesiates **9** and **10**. Obtained as crystalline solids in yields of 76 % and 63 % respectively, these can be described as lower order ates, in the sense that their alkali metal to magnesium ratio is 1:1; whereas higher order ates would have 2:1 ratios. Note that these structures were formed independent of the starting stoichiometry as reaction mixtures that have 3:1 amide/*n*Bu ratios, as in [Na_4_Mg_2_(TMP)_6_(*n*Bu)_2_], still afforded **9** and **10** as the main products. To the best of our knowledge these are the first alkali metal magnesiates of 2,6‐diisopropyl‐*N*‐(trimethylsilyl)aniline to be synthesised, isolated from solution and crystallographically characterised (vide infra). However, sodium and potassium Group 13 ate complexes are known for aluminium.^20^, 2

### Crystallographic characterisation

Tables S7 and S8 in the Supporting Information list the crystal data for all ten new compounds, while Table S9 compares selected metric data across the series.

**Figure 2:**
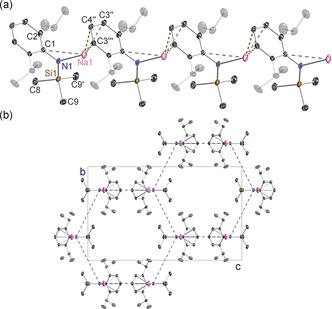
a) Infinite linear chain structure of [Na{N(SiMe_3_)(Dipp)}]_∞_ (**1**), showing atomic connectivity between the metal and the ligand. b) Packing diagram of complex **1** (viewed along *a*‐axis). Thermal ellipsoids are displayed at 35 % probability and hydrogen atoms have been omitted for clarity. The dashed lines represent short Na⋅⋅⋅C contacts. Symmetry operation to generate equivalent atoms denoted ′: *x*, −*y*+1/2, *z*; ′′: *x*−1, *y*, *z*; ′′′: *x*−1, −*y*+1/2, *z*. Selected bond lengths [Å] and angles [°]: Na1−N1 2.2585(16); Na1−C4′′ 2.696(2); Na1−C3′′′ 2.7514(16); Na1−C1 3.1279(18); Si1−N1 1.6816(16); N1−C1 1.381(2); C1−C2 1.4260(17); N1‐Na1‐C4′′ 130.07(6); N1‐Na1‐C3′′′ 137.70(5); C1‐N1‐Si1 123.82(11); C1‐N1‐Na1 116.41(11); Si1‐N1‐Na1 119.77(8); N1‐C1‐C2 121.69(8).

**Figure 3:**
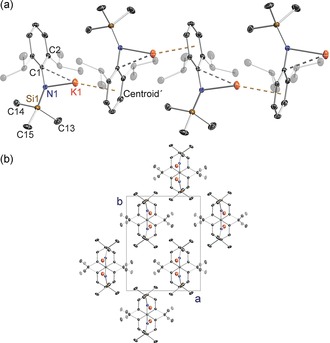
a) Infinite zig‐zag chain structure of [K{N(SiMe_3_)(Dipp)}]_∞_ (**2**), showing atomic connectivity between the metal and the ligand. b) Packing diagram of complex **2** (viewed along *c*‐axis). Thermal ellipsoids are displayed at 35 % probability and hydrogen atoms have been omitted for clarity. The dashed lines represent short K⋅⋅⋅C contacts. Symmetry operation to generate equivalent atoms denoted ′: *x*, −*y*+3/2, *z*−1/2. Selected bond lengths [Å] and angles [°]: K1−N1 2.6755(12); K1−C1 3.0735(14); K1−Centroid′ 2.81; Si1−N1 1.6615(14); N1−C1 1.355(2); C1−C2 1.445(2); C1‐N1‐Si1 137.47(10); C1‐N1‐K1 93.59(8); Si1‐N1‐K1 128.62(7); N1‐K1‐Centroid′ 154.53.

In the absence of interrupting donor molecules, the parent sodium amide **1** and potassium amide **2** adopt infinite chain structures, though each has a distinctive propagation (Figures 2 and 3, respectively). The former has a linear arrangement with the amido substituents *syn* with respect to the [ML]_*n*_ chain and hence eclipsing each other along the chain, whereas the latter has an *anti* conformation with the Dipp substituent on one molecular unit facing the SiMe_3_ substituent of the adjacent unit. Structure **1** is a channel solvate with both the solvent channels and the one‐dimensional polymers propagating parallel to the *a* direction. For **2** the one‐dimensional polymeric chains propagate parallel to *c* and neighbouring chains link through Me⋅⋅⋅K agostic interactions. Such interchain agostic interactions are not observed in **1**. The strongest metal–ligand bond in each case is to the amido N atom (Na1−N1 2.2585(16) Å; K1−N1 2.6755(12) Å), but as these can only provide the alkali metal with a coordination number of one, the Dipp substituents also engage with the metal. The propagating interactions between monomeric units in **1** are Na contacts to the *para* and *meta*/*meta′* Dipp C atoms (2.696(2) and 2.7514(16) Å) making the Dipp unit η^3^‐bound, though there is an additional intramolecular Na1⋅⋅⋅C1 contact (3.1279(18) Å) to the *ipso*‐C atom within the monomeric unit. Possessing the larger, softer K cation, **2** propagates through η^6^‐C−K engagement (centroid length, 2.81 Å; range of K−C lengths, 3.1014(14)–3.1647(16) Å) and there is an additional close intramonomeric contact (K−C1, 3.0735(14) Å). The N1‐K1‐centroid′ angle is 154.53° reflecting the bulky heteroleptic nature of the substituents attached to the amido nitrogen atom. Crystallographically characterised polymeric sodium and potassium amides are comparatively rare.^21^, 2, 3

**Figure 4:**
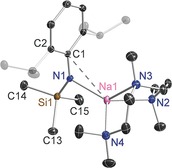
Molecular structure of [Na{N(SiMe_3_)(Dipp)}(PMDETA)] (**4**). Thermal ellipsoids are displayed at 35 % probability and hydrogen atoms have been omitted for clarity. The dashed lines illustrate the Na⋅⋅⋅C contact. Selected bond lengths [Å] and angles [°]: Na1−N1 2.3206(12); Na1−N2 2.4862(13); Na1−N3 2.4188(14); Na1−N4 2.5201(14); Na1−C1 3.0937(14); Si1−N1 1.6565(12); N1−C1 1.3729(17); C1−C2 1.4321(19); N1‐Na1‐N2 125.66(5); N1‐Na1‐N3 125.63(5); N3‐Na1‐N2 75.39(4); N1‐Na1‐N4 123.87(5); N3‐Na1‐N4 74.71(5); N2‐Na1‐N4 109.45(5); C1‐N1‐Si1 140.09(10); C1‐N1‐Na1 111.17(8); Si1‐N1‐Na1 107.98(6); N1‐C1‐C2 121.51(12).

PMDETA deaggregates the polymer of **1** all the way down to a monomer within **4** (Figure 4). Accompanied by the amido N, the tridentate solvent surrounds the Na centre within a distorted (N×4) tetrahedron in which the shortest bond is to the amido N atom (Na−N1, 2.3206(12) Å) with the PMDETA bonds to Na spanning the range 2.4188(14)–2.5201(14) Å. There is an additional Na⋅⋅⋅*ipso‐*C contact (Na−C1, 3.0937(14) Å), presumably an unavoidable artefact of the short Na−N1 bond, though this is longer than that in polymeric **1**, intimating that PMDETA is more effective than the η^3^‐bound Dipp at binding to Na, a point reinforced by an increased C1‐N1‐Si1 bond angle (140.09° cf., 123.85(19)° in **2**). As reported by Westerhausen, less bulky sodium amides can still dimerise in the presence of PMDETA in [{Na(NPh_2_)(PMDETA)}_2_].21i, 4

**Figure 5:**
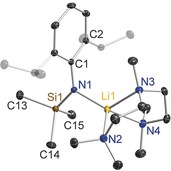
Molecular structure of [Li{N(SiMe_3_)(Dipp)}(PMDETA)] (**3**). Thermal ellipsoids are displayed at 35 % probability and hydrogen atoms have been omitted for clarity. Selected bond lengths [Å] and angles [°]: Li1−N1 2.020(3); Li1−N2 2.314(3); Li1−N3 2.148(3); Li1−N4 2.216(3); Si1−N1 1.6813(14); N1−C1 1.3898(19); C1−C2 1.429(2); N1‐Li1‐N2 122.13(13); N1‐Li1‐N3 125.62(14); N1‐Li1‐N4 122.30(14); N3‐Li1‐N4 84.00(11); N3‐Li1‐N2 83.16(10); N4‐Li1‐N2 108.19(13); C1‐N1‐Si1 128.77(11); C1‐N1‐Li1 115.86(13); Si1‐N1‐Li1 114.96(10); N1‐C1‐C2 122.22(14).

Unsurprisingly, the PMDETA lithium congener **3** is also monomeric (Figure 5) with a short Li1−(amido)N1 bond (2.020(3) Å) and three longer bonds to PMDETA N atoms (Li1−N2 2.314(3); Li1−N3 2.148(3); Li1−N4 2.216(3); mean value, 2.226 Å). Note that **3** and **4** are isomorphic and isostructural. The more crowded distorted tetrahedral environment about the smaller Li compared to that of Na in **4** is reflected in a less obtuse C1‐N1‐Si1 bond angle (128.77(11)°). Andrews has reported a monomeric lithium amide PMDETA complex in [Li(NPhMe)(PMDETA)].^22^, 5

**Figure 6:**
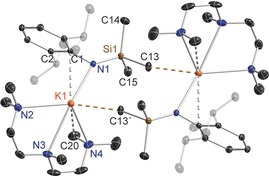
Molecular structure of [{K{N(SiMe_3_)(Dipp)}(PMDETA)}_2_] (**5**). Thermal ellipsoids are displayed at 35 % probability and hydrogen atoms have been omitted for clarity. The dashed lines illustrate the K⋅⋅⋅C contacts. Symmetry operation to generate equivalent atoms denoted ′: −*x*+1, −*y*, −*z*. Selected bond lengths [Å] and angles [°]: K1−N1 2.7174(13); K1−N2 2.9203(15); K1−N3 2.9306(15); K1−N4 2.8230(15); K1−C1 2.9806(14); K1−C13′ 3.2272(17); K1−C20 3.2166(2); Si1−N1 1.6569(13); N1−C1 1.3716(18); C1−C2 1.432(2); N1‐K1‐N4 99.71(4); N1‐K1‐N2 127.56(4); N4‐K1‐N2 115.64(5); N1‐K1‐N3 157.12(4); N4‐K1‐N3 60.85(4); N2‐K1‐N3 60.58(4); N1‐K1‐C1 27.36(4); N1‐K1‐C13′ 90.96(4); C1‐K1‐C20 98.760(3); C1‐K1‐C13′ 109.373(3); C1‐N1‐Si1 133.05(10); C1‐N1‐K1 87.06(8); Si1‐N1‐K1 139.87(7); N1‐C1‐C2 122.09(13).

A single tridentate PMDETA ligand is not sufficient to coordinatively saturate the K centre of the amide **5**, which therefore exists as a centrosymmetric dimer (Figure 6). Dimerisation is manifested by a long K1−C13′ intermolecular interaction (3.2272(17) Å) involving a methyl of the SiMe_3_ group,^23^ which closes an eight‐membered (KNSiC)_2_ ring similar to that found by Coles and Cloke in [K(N{SiMe_2_(C_6_H_4_‐2‐OMe)}_2_)] with an analogous K−C intermolecular interaction of 3.222(2) Å.^24^ The primary K coordination sphere of **5** is composed of four N atoms, with the shortest bond being to the amido N1 (2.7174(13) Å) with those to PMDETA (N2, N3, N4) having a mean length of 2.8913 Å. This intramolecular tetranitrogen coordination brings K into close proximity to both the *ipso‐*C1 of the Dipp substituent and one terminal Me of the PMDETA ligand (lengths of 2.9806(14) Å and 3.2166(2) Å, respectively). Small PMDETA bite angles dictate that this KN_4_ coordination sphere is far removed from a perfect tetrahedron (range of bond angles, 60.58(4)° to 127.56(4)°; mean 103.57°).6

**Figure 7:**
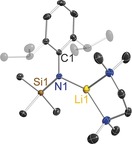
Molecular structure of [Li{N(SiMe_3_)(Dipp)}(TMEDA)] (**6**). Thermal ellipsoids are displayed at 35 % probability, hydrogen atoms, and the disordered component of TMEDA ligand and two methyl groups of one *i*Pr unit are omitted for clarity. Selected bond lengths [Å] and angles [°]: Li1−N1 1.905(4); N1−C1 1.388(2); Si1−N1 1.6756(17); Si1‐N1‐Li1 121.50(15); C1‐N1‐Li1 108.20(17); C1‐N1‐Si1 130.30(13).

Only with the lithium amide is a monomeric structure retained upon substituting PMDETA by the smaller TMEDA ligand. The resulting structure, **6**, is shown in Figure 7. There is disorder present which somewhat compromises the Li−N (TMEDA) metric data, but the primary coordination about Li is unequivocal comprising three nitrogen atoms—one amido and two TMEDA types.7

**Figure 8:**
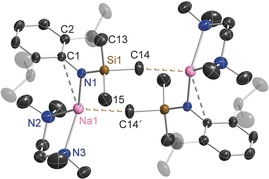
Molecular structure of [{Na{N(SiMe_3_)(Dipp)}(TMEDA)}_2_] (**7**). Thermal ellipsoids are displayed at 30 % probability and hydrogen atoms have been omitted for clarity. Dashed lines illustrate the Na⋅⋅⋅C contacts. Symmetry operation to generate equivalent atoms denoted ′: −*x*+1, −*y*+2, −*z*+1. Selected bond lengths [Å] and angles [°]: Na1−N1 2.2847(14); Na1−N2 2.4726(17); Na1−N3 2.461(2); Na1−C14′ 2.869(2); Na1−C1 2.9379(16); Si1−N1 1.6631(13); N1−C1 1.3785(19); C1−C2 1.424(2); N1‐Na1‐N3 133.21(7); N1‐Na1‐N2 130.49(6); N3‐Na1‐N2 75.38(6); N1‐Na1‐C14′ 103.78(6); N3‐Na1‐C14′ 106.10(7); N2‐Na1‐C14′ 103.19(7); C1‐N1‐Si1 132.08(11); C1‐N1‐Na1 103.88(9); Si1‐N1‐Na1 124.02(7); N1‐C1‐C2 121.54(14).

In contrast the larger sodium centre in **7** cannot be coordinatively satisfied by the amide/TMEDA combination so akin to the case of the K PMDETA complex **5** dimerisation takes place through Na⋅⋅⋅C−Si interactions to close a (NaNSiC)_2_ ring (Figure 8). Na approaches the anionic amido N atom more closely (2.2847(14) Å) than the neutral TMEDA N atoms (mean length, 2.4668 Å), giving rise to a trigonal pyramidal primary NaN_3_ coordination (sum of bond angles, 339.08°), but as in **5** this crowded coordination brings the metal into close proximity with intramolecular C1 (2.9379(16) Å) and intermolecular C14′ (2.869(2) Å), making it five‐coordinate overall.8

**Figure 9:**
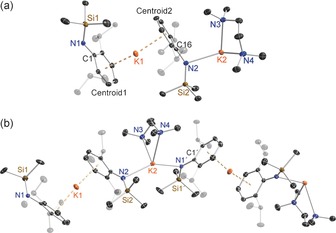
a) Asymmetric unit of the structure of [{K{N(SiMe_3_)(Dipp)}}_2_(TMEDA)]_∞_ (**8**). b) Section of extended framework structure showing atom connectivity between the metal and the [N(SiMe_3_)(Dipp)] ligand. Thermal ellipsoids are displayed at 35 % probability and hydrogen atoms have been omitted for clarity. Dashed lines denote the K⋅⋅⋅aryl contacts. Symmetry operation to generate equivalent atoms denoted ′: *x*+1/2, −*y*+2, *z*. Selected bond lengths [Å] and angles [°]: K1−Centroid1 2.7943(8); K1−Centroid2 2.7909(8); N1−C1 1.354(4); N1−Si1 1.663(3); Si2−N2 1.651(3); K2−N3 2.780(3); K2−N1′ 2.830(3); K2−N2 2.831(3); K2−N4 2.932(3); N2−C16 1.350(4); Centroid1‐K1−Centroid2 165.8; C1‐N1‐Si1 135.1(2); C1′‐N1′‐K2 112.00(19); Si1′‐N1′‐K2 110.56(12); N3‐K2‐N1′ 93.22(9); N3‐K2‐N2 103.45(9); N1′‐K2‐N2 148.95(9); N3‐K2‐N4 66.72(9); N1′‐K2‐N4 97.86(9); N2‐K2‐N4 112.70(9); C16‐N2‐Si2 143.5(2); C16‐N2‐K2 108.7(2); Si2‐N2‐K2 107.61(13).

The crystallographic determination of potassium amide **8** (Figure 9) established it as a hemi‐TMEDA solvate though the reaction was carried out using a 1:1 K/TMEDA stoichiometric ratio. Note that this structure contains two crystallographically independent molecules within the asymmetric unit cell, with slightly different structural parameters, but for brevity only one is discussed here. Because of this donor deficiency, **8** exhibits an infinite zig‐zag chain structure, propagating parallel to the *a* direction, with two distinctly different K environments. K2 occupies a highly distorted tetrahedral N_4_ site [bond angles ranging from 66.72(9)° to 148.95(9)°, with the extremities representing the TMEDA bite angle and amido‐K‐amido angle, respectively] comprising the two TMEDA N atoms and two anionic N atoms, belonging to two amide ligands (range of bond lengths, 2.780(3) to 2.830(3) Å). In contrast, K1 lies between the aryl rings of two *transoid* disposed Dipp ligands, in a near linear sandwich arrangement (centroid‐K‐centroid angle, 165.8°; mean K−C(centroid) length, 2.7926 Å) architecturally akin to classic structures such as bis(benzene)chromium, though the bonding in potassium sandwiches is electrostatic in origin.15c, 25 Formally this [(K)^+^(R_2_K⋅TMEDA)^−^] formulation of **8** in which K1 does not engage with an anionic centre (discounting any delocalisation within the Dipp aryl ring) can be described as a potassium potassiate. Though rare, potassium potassiate structures have been reported previously.^26^, 9

**Figure 10:**
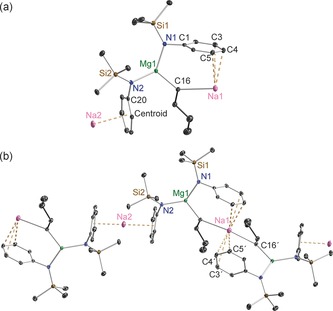
a) Asymmetric unit of the structure of [NaMg{N(SiMe_3_)(Dipp)}_2_(μ‐*n*Bu)]_∞_ (**9**). Note that Na1 and Na2 are at half occupancy. b) Section of extended framework structure showing atomic connectivity between the metals, *n*‐butyl and connecting N atom of the [N(SiMe_3_)(Dipp)] ligands. Thermal ellipsoids are displayed at 35 % probability and hydrogen atoms and *i*Pr groups have been omitted for clarity. The dashed lines illustrate the Na⋅⋅⋅aryl contacts. Symmetry operation to generate equivalent atoms denoted ′: −*x*+1, *y*, −*z*+1/2. Selected bond lengths [Å] and angles [°]: Na1−C16 2.779(2); Na1−C4 2.793(2); Na1−C5 2.913(2); Na1−C3 3.2535(20); Na2−Centroid 2.5311(1); Mg1−N1 2.0263(16); Mg1−N2 2.0393(15); Mg1−C16 2.1533(19); Si1−N1 1.7030(16); Si2−N2 1.7042(16); N1−C1 1.414(2); N2−C20 1.405(2); centroid‐Na2‐centroid 180.0; Mg1‐C16‐Na1 126.94(8); C16′‐Na1‐C16 132.53(9); N1‐Mg1‐N2 129.36(7); N1‐Mg1‐C16 112.50(7); N2‐Mg1‐C16 117.83(7); C1‐N1‐Si1 120.85(12); C1‐N1‐Mg1 105.48(11); Si1‐N1‐Mg1 133.55(9); C20‐N2‐Si2 122.86(11); C20‐N2‐Mg1 109.11(11); Si2‐N2‐Mg1 127.99(8).

**Figure 11:**
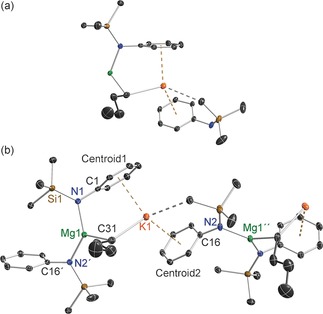
a) Structure of [KMg{N(SiMe_3_)(Dipp)}_2_(μ‐*n*Bu)]_∞_ (**10**), showing the contents of the asymmetric unit. b) Section of extended framework structure showing atomic connectivity between the metals, *n*‐butyl and connecting N atom of the [N(SiMe_3_)(Dipp)] ligands. Thermal ellipsoids are displayed at 35 % probability, hydrogen atoms, *i*Pr groups, one disordered component of a ‐SiMe_3_ group and one disordered methylcyclohexane molecule of crystallisation have been omitted for clarity. The dashed lines illustrate the K⋅⋅⋅aryl contacts. Symmetry operation to generate equivalent atoms denoted ′: *x*, −*y*+1, *z*−1/2; ′′: *x*, −*y*+1, *z*+1/2. Selected bond lengths [Å] and angles [°]: K1−C31 3.046(3); K1−Centroid1 2.9162(1); K1−Centroid2 2.9239(1); Mg1−N1 2.0327(18); Mg1−N2′ 2.0341(17); Mg1−C31 2.150(2); Si1−N1 1.7092(17); N1−C1 1.415(2); N2−C16 1.411(2); N2−Mg1′′ 2.0340(17); Centroid1‐K1‐Centroid2 147.4; C31‐K1‐Centroid1 91.943(3); C31‐K1‐Centroid2 117.537(2); Mg1‐C31‐K1 94.26(9); N1‐Mg1‐N2′ 133.18(7); N1‐Mg1‐C31 110.36(9); N2′‐Mg1‐C31 116.34(9); C1‐N1‐Si1 121.87(13); C1‐N1‐Mg1 111.36(12); Si1‐N1‐Mg1 126.69(9); C16‐N2‐Mg1′′ 122.16(13).

**Figure 12:**
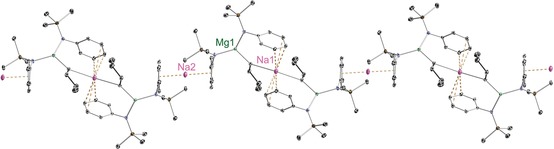
Part of the polymeric helical chain of [NaMg{N(SiMe_3_)(Dipp)}_2_(μ‐*n*Bu)]_∞_ (**9**) that propagates parallel to the crystallographic *c*‐axis.

**Figure 13:**
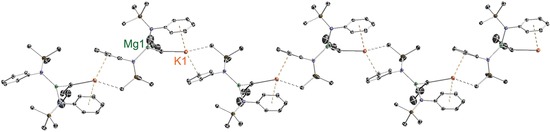
Part of the polymeric helical chain of [KMg{N(SiMe_3_)(Dipp)}_2_(μ‐*n*Bu)]_∞_ (**10**) propagating parallel to the *c*‐axis.

Magnesiates **9** and **10**, which both have a 2:1 amido/butyl stoichiometric ratio different to the 3:1 ratio in the aforementioned pre‐inverse‐crown template base [Na_4_Mg_2_(TMP)_6_(*n*Bu)_2_], were expected to adopt different structural architectures from this TMP derivative. X‐ray crystallographic determinations duly confirmed this expectation. The salient difference is that both **9** and **10** have infinite helical chain structures (Figures 10–13) and not the ring architecture that appears to be the key feature behind the special templating metallation ability of [Na_4_Mg_2_(TMP)_6_(*n*Bu)_2_].^8^, 10, 11

Sodium magnesiate **9** displays two distinct types of (half occupancy) Na atom separated by a magnesiate anion, neither of which engage with the amido N atom. Na1 lies upon a twofold rotation axis and bonds to two symmetrically equivalent butyl C atoms (C16/C16′, 2.779(2) Å) and two symmetrically equivalent aryl groups (C4/C4′ and C5/C5′) at the *para*/*meta* positions (2.793(2) Å and 2.913(2) Å, respectively), making it six‐coordinate overall. The next shortest distance between Na1 and the aryl group is to C3 (at 3.2535(20) Å), which seems too long, so the hapticity of the aryl substituent is best regarded as η^2^. Lying on a crystallographic inversion centre sandwiched between two aryls from neighbouring magnesiate ions, Na2 binds in a η^6^ arrangement to both of them (range of Na2−C bond lengths, 2.684(2)–3.1565(18) Å; Na−centroid distance 2.5311(1) Å; centroid‐Na‐centroid angle, 180.0°). Propagation of this contacted ion pair structure into an infinite helical chain (Figure 12) is through these distinct types of Na−C interaction. Mg1 occupies a N_2_C distorted trigonal planar coordination (sum of bond angles; 359.69°) comprising two amide ligands (mean Mg−N distance, 2.0328 Å) and one butyl ligand (Mg1−C16, 2.1533(19) Å). The helical chain structure of **10** (Figure 13) is less intricate than that of **9** as it contains only one alkali metal (K) site. Sandwiched between two aryl rings (K1−centroid1 2.9162(1) Å; K1−centroid2 2.9239(1) Å; centroid1‐K1‐centroid2, 147.4°), K1 also interacts with the α‐C of the Bu ligand (K1−C31 3.046(3) Å), while there is a contact with one of the CH_3_(Si) groups though the disorder in this group negates an accurate bond length. Consistent with the interaction between the alkali metal and the magnesiate ion being predominately electrostatic in nature the dimensions of the Mg1 centre in **10** (mean Mg−N, 2.0334 Å; Mg−C31, 2.150(2) Å; sum of bond angles subtending Mg1, 359.82°) match those in the sodium magnesiate 12.13

### Solution NMR spectroscopic characterisation

All the new compounds **1**–**10** were also characterised in solution by ^1^H and ^13^C{^1^H} NMR spectroscopy recorded at ambient temperature. Assignments were aided by ^1^H,^1^H‐COSY and ^1^H,^13^C‐HSQC experiments. In general, the data obtained (see the full experimental details in the Supporting Information) supported the formulae established by X‐ray crystallography of the solid samples.

Since the polymeric solids **1** and **2** were poorly soluble in arene solvents, their solutions were made up in a mixture of C_6_D_6_ and [D_8_]THF solvents. This meant we were viewing THF solvates instead of the pure, unsolvated parent amides. All the expected resonances were observed and irrespective of whether the alkali metal was sodium or potassium the chemical shifts essentially matched (e.g., the quaternary C atom bound to N was located at 157.5 ppm and 156.8 ppm in the ^13^C spectra of **1** and **2**, respectively), giving credence to the view that the bonding between the amide and alkali metal is predominately electrostatic. The presence of PMDETA in **3**–**5** enhanced the solubility of the amides such that they could dissolve well in C_6_D_6_ without the addition of polar THF. The ^7^Li NMR spectrum of **3** revealed a single species at 25 °C. Its ^1^H NMR spectrum showed one methine C*H* resonance and two distinct Me resonances for the Dipp substituent, consistent with one type of amido group. The aforementioned quaternary C atom bound to N appears at 157.5 ppm in the ^13^C spectrum, essentially identical to those in the THF solvates of **1** and **2**, and to those in the PMDETA solvates of the sodium and potassium amide **4** and **5** (at 157.8 ppm and 157.0 ppm, respectively). There are differences but surprisingly only modest ones for the PMDETA resonances across the series **3**–**5**. For example, the *C*H_3_ resonances appear at 46.0/44.9 ppm, 45.3/43.9 ppm, and 44.9/40.9 ppm for terminal/central positions respectively in the ^13^C NMR spectra. Both the Li and Na TMEDA solvates **6** and **7**, being molecular solids, dissolve in C_6_D_6_, in contrast to the polymeric hemi‐TMEDA solvate **8**, which requires some [D_8_]THF to exhibit solubility in the arene. Consistent with the monomeric structure in the crystal, the ^7^Li NMR spectrum of **6** shows a solitary resonance. TMEDA is bound to the Li as evidenced by the chemical shift and order of the TMEDA resonances in the ^1^H NMR spectrum (Me, 1.61 ppm; C*H*_2_, 1.44 ppm), which bear a close resemblance to those in the Na TMEDA solvate (1.57 ppm and 1.49 ppm, respectively). As anticipated, the mixed solvent solution of **8** contains free TMEDA, with the order of its resonances reversed in comparison (C*H*_2_, 2.12 ppm; Me, 1.97 ppm), as [D_8_]THF will now be solvating the larger alkali metal.

**Scheme 3:**
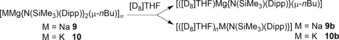
Breakdown of the complexes **9** and **10** in [D_8_]THF.

Magnesiates **9** and **10** conform to the pattern seen with the other polymeric solids **1**, **2** and **8** in requiring the addition of [D_8_]THF for their dissolution in C_6_D_6_. Hence, the solutions will contain THF solvates. The reduced solubility of the donor‐free compounds in hydrocarbon solvents such as methylcyclohexane immediately signalled that we were dealing with structures distinct from that of the cyclic hexanuclear sodium magnesiate [Na_4_Mg_2_(TMP)_6_(*n*Bu)_2_], which shows excellent solubility in hydrocarbon media. The ^1^H and ^13^C NMR spectra of **9** show two distinct types of amido group. For example, the former shows two *p*‐C*H*‐Ar triplet resonances at 6.85 and 6.64, two C*H*(CH_3_)_2_ septets at 4.27 and two Si(C*H*_3_)_3_ singlets at 0.30 ppm and 0.25 ppm. This could be due to two distinct amido groups within the same molecule or alternatively two distinct amido‐containing molecules. The chemical shift of the C*H*_2_‐*n*Bu multiplet appearing at −0.26 ppm indicates that this ligand is more likely to be attached to Mg as it would appear more upfield if attached to the more electropositive metal Na {for example, the same resonance appears in C_6_D_6_ solutions of [{Mg(TMP)(*n*Bu)}_2_] at 0.05 ppm^27^ and [Mg(TMP)(*n*Bu)⋅IPr] (IPr=1,3‐bis‐(2,6‐diisopropylphenyl)imidazol‐2‐ylidene) at −0.42 ppm}.^28^ Due to the ambiguity of these spectra we turned to diffusion‐ordered spectroscopy (DOSY)^29^ to attempt to find a resolution. For an accurate molecular weight (MW) determination we utilised the external calibration curve (ECC) approach with normalised diffusion coefficients that has recently been developed by the Stalke group.^30^ This novel approach takes into account the shape of the molecule allowing accurate MW predictions with a maximum error of less than 9 %. The beauty of this approach is that only one internal reference (that also can be the solvent) is necessary, whereas previous approaches required multiple references. In this case tetramethylsilane was employed. DOSY determined MWs of species in solution were estimated using the diffusion coefficient values for **9** in [D_8_]THF (Figure S1, Tables S2–S3 in the Supporting Information). Two distinct diffusion coefficients were obtained consistent with two distinct species. The best fit for these were found to be the homometallic THF solvates [([D_8_]THF)_*x*_Mg{N(SiMe_3_)(Dipp)}(μ‐*n*Bu)] **9 a** (surprisingly the error when *x*=1, −6 %, was found to be much less than that for *x*=2, −21 %, a result at odds with the aforementioned crystal structure, which contained two THF ligands) and [([D_8_]THF)_2_Na{N(SiMe_3_)(Dipp)}] **9 b** with the error from their calculated MWs being −6 % and −7 %, respectively. Significantly the heterometallic ate **9** would have an error of 36 % if unsolvated, or 43 % if solvated by one THF ligand. Clearly the magnesiate that crystallises from hydrocarbon/arene solution breaks down to homometallic species in the presence of the strong Lewis base THF (Scheme 3).^31^ Organomagnesium compounds are synonymous with redistribution reactions, most notably the Schlenk equilibrium. Unsurprisingly, the potassium magnesiate **10** was found to undergo the same disproportionation reaction in [D_8_]THF (Scheme 3), with the corresponding DOSY data consistent with the two homometallic species [([D_8_]THF)Mg{N(SiMe_3_)(Dipp)}(μ‐*n*Bu)] **10 a** and [([D_8_]THF)K{N(SiMe_3_)(Dipp)}] **10 b** with small errors of −4 % and +1 %, respectively, versus the calculated MWs. Supporting evidence that **9 a** and **10 a** are the same solution species comes from the close similarity of their chemical shifts [for example: ^1^H, C*H*_2_‐*n*Bu at −0.26 ppm and −0.22 ppm, respectively; Si(C*H*_3_)_3_ at 0.30 ppm in both; ^13^C, *C*H_2_‐*n*Bu at 9.3 ppm and 9.0 ppm, respectively; (*o*‐*C*_q_‐Ar) appearing at 145.1 ppm in both]. Ruhlandt‐Senge also reported [(THF)_2_Mg{N(SiMe_3_)(Dipp)}(*n*Bu)] and NMR data are consistent with that found in **9 a** and **10 a** (e.g., C*H*_2_‐*n*Bu at 9.4 ppm in neat C_6_D_6_).3

### Reactivity studies

Our failure to synthesise a template ring complex as [Na_4_Mg_2_(TMP)_6_(*n*Bu)_2_] incorporating the arylsilyl amido ligand [N(SiMe_3_)(Dipp)] in place of TMP reduced our expectation of realising enhanced or special reactivities, but for completeness we carried out some representative metallation–iodination reactions. Table S10 in the Supporting Information compiles those carried out between 4,4‐dimethyl‐2‐phenyl‐oxazoline (**12 a**) and each of the following complexes: the solvent‐free parent sodium and potassium amides **1** and **2**; the heavier alkali metal magnesiates **9** and **10**; and for comparison, the alkylmagnesium amide [Mg{N(SiMe_3_)(Dipp)}(*n*Bu)] (**Mg**). Table S11 lists the reactions between *N*,*N*‐diisopropylbenzamide (**12 b**) and the same set of potential bases. In both cases the attempted metallations were performed in methylcyclohexane and the subsequent iodine quenches were done in THF. Full details are provided in the Experimental Section.

Owing mainly to their poor hydrocarbon solubility, unsolvated **1** and **2** proved unreactive with both of the aromatic substrates even under reflux conditions. Though magnesium bases are generally significantly less reactive than alkali metal bases, heteroleptic [Mg{N(SiMe_3_)(Dipp)}(*n*Bu)] proved effective at deprotonating the oxazoline substrate (the iodo product **13 a** was quantitative when the metallation was performed under reflux conditions), since it contains a butyl anion as well as an amide and is more soluble in hydrocarbon media. The magnesium base also metallated the slightly more challenging benzamide substrate, but much less effectively (yield of iodo product **13 b**, 42 %). In both cases the metallation–iodination operation took place regioselectively at the *ortho* position in keeping with the directed *ortho*‐metallation (D*o*M) principle.^9^ Interestingly, when the alkylmagnesium amide is incorporated within the sodium and potassium ates **9** and **10**, the reactivity towards **12 a** diminishes especially when the metallation is performed at room temperature dropping from 80 % to 10 % and 11 %, respectively. Rerunning the metallations under reflux conditions greatly improves the yields of the iodo product **13 a**, though they still fall short of that obtained by [Mg{N(SiMe_3_)(Dipp)}(*n*Bu)]. Only when the amount of ate base is doubled do the yields obtained approach 100 %. Yields of the iodo product **13 b** from reaction of **9** or **10** with the benzamide never reach quantitative even at reflux temperature with the best just over 50 %.

Attempts were made to gain insight into the intermediate metallated complexes prior to the iodination step. A modicum of success was made in the reaction between “sodium magnesiate **9**” and the oxazoline **12 a**. The reaction solution deposited a crystalline solid in the magnesium complex [Mg{N(SiMe_3_)(Dipp)}R] (**11**), in which R is *ortho*‐deprotonated 4,4‐dimethyl‐2‐phenyl‐oxazoline. Unfortunately X‐ray crystallographic studies of **11** revealed a highly disordered structure that negates its inclusion here, though NMR studies confirmed its formula. Though monometallic **11** could result from a disproportionation of bimetallic ate **9**, it is possible that **9** never formed in the hydrocarbon medium, but was in fact a mixture of **1** and [Mg{N(SiMe_3_)(Dipp)}(*n*Bu)], with **11** forming as a result of the latter deprotonating the oxazoline substrate. However, given that the yield of iodo product **12 a** using [Mg{N(SiMe_3_)(Dipp)}(*n*Bu)] on its own was 80 % at 25 °C, but is only 10 % using **9** as the base under the same conditions, this suggests that the presence of the sodium amide component significantly inhibits reactivity of the alkylmagnesium amide. Since it was clear that these magnesiates do not possess robust bimetallic structures like that of the cyclic hexanuclear sodium magnesiate [Na_4_Mg_2_(TMP)_6_(*n*Bu)_2_], it was deemed not worthwhile to explore their reactivity any further.

## Conclusion

This study has uncovered a remarkable s‐block complex structural diversity based on the amide [N(SiMe_3_)(Dipp)]^−^ functioning as a ligand on its own or in combination with an *n*‐butyl ligand. The parent sodium and potassium amides adopt infinite chain structures, with linear or zig‐zag arrangements, respectively, adding to the relatively few known crystallographically characterised unsolvated s‐block metal amides. Tridentate PMDETA deaggregates the sodium polymer to a monomer and also generates a monomeric lithium amide, but it is not sufficient to coordinatively saturate the K centre of the amide, which exists as a centrosymmetric dimer, with dimerisation expressed by a long K−CH_3_SiMe_2_ intermolecular interaction that closes an eight‐atom (KNSiC)_2_ ring. Reducing the chelating capability of the donor solvent via TMEDA has little effect on the lithium amide, which remains monomeric, but the sodium amide dimerises by means of Na⋅⋅⋅C−Si interactions mimicking the case of the PMDETA potassium amide. Refusing half a molar equivalent of TMEDA despite a 1:1 K/TMEDA stoichiometric ratio in the solution, the potassium amide crystallises as a hemi‐TMEDA solvate. Its infinite chain structure has distinct K coordination environments, one occupying a N_4_ site, the other sandwiched between aryl rings of two Dipp ligands, classifies it as a novel potassium potassiate.

Both crystalline sodium and potassium magnesiates display a 2:1 amido/butyl stoichiometric ratio in infinite helical chain structures conflicting with the 3:1 ratio of the template base [Na_4_Mg_2_(TMP)_6_(*n*Bu)_2_], which inspired this study. Lack of hydrocarbon solubility denied any opportunity for these ates to display special reactivities like that of the template base. THF was needed for solubility but DOSY studies indicate that the donor promotes fragmentation of the magnesiates into homometallic moieties. The ring architecture is the key feature behind the special templating metallation ability of [Na_4_Mg_2_(TMP)_6_(*n*Bu)_2_], but such architectures proved inaccessible with the [N(SiMe_3_)(Dipp)] ligand. Despite this disappointment the study is important, as it has clearly established that the presence of aryl groups can be an inhibiting factor to molecular ring formation as their π‐faces can engage intermolecularly with alkali metals to help construct polymeric chains. Future work will focus on circumventing this solubility/structural problem by using bulky lipophilic non‐aryl ligands.

## Experimental Section

**General procedures**: All reactions and manipulations were performed under a protective atmosphere of dry pure argon gas using standard Schlenk tube or glovebox techniques. Solvents were dried by heating to reflux over sodium benzophenone ketyl and distilled under nitrogen prior to use. Methylcyclohexane was distilled over sodium metal and stored with molecular sieves (4 Å). Deuterated NMR solvents were degasified and stored over molecular sieves (4 Å) prior to use. 2,6‐diisopropylaniline, *N*,*N*,*N′*,*N′′*,*N′′*‐pentamethyldiethylenetriamine (PMDETA) and *N*,*N*,*N′*,*N′*‐tetramethylethylenediamine (TMEDA) were purchased from Aldrich, dried by heating to reflux over calcium hydride and stored with molecular sieves (4 Å) under nitrogen prior to use. *n*BuLi (1.6 m in *n*‐hexane) and *n*Bu_2_Mg (1 m in *n*‐heptane) solutions were purchased from Aldrich and titrated prior to use. Trimethylsilyl chloride, tetramethylsilane, sodium *tert*‐butoxide, potassium *tert*‐butoxide, 4,4‐dimethyl‐2‐phenyl‐oxazoline, *N*,*N*‐diisopropylbenzamide and 1,10‐Phenanthroline were purchased from Aldrich and used as received. *n*BuNa,^32^ K(CH_2_SiMe_3_)^33^ and [N(H)(SiMe_3_)(Dipp)]^34^ (Dipp=2,6‐*i*Pr_2_‐C_6_H_3_) were prepared according to literature procedures. NMR spectra were recorded on a Bruker DPX 400 NMR spectrometer, operating at 400.13 MHz for ^1^ H, 155.5 MHz for ^7^Li and 100.6 MHz for ^13^C. ^1^H and ^13^C{^1^H} spectra were referenced to the appropriate solvent signal, ^7^ Li NMR spectra were referenced against LiCl in D_2_O at 0.00 ppm. Elemental analyses of the compounds **1**–**10** were carried out using a PerkinElmer 2400 elemental analyser. Full characterisation details are given in the Supporting Information.

### Reactivity studies

**Isolation and characterisation of metallated intermediate 11**: 
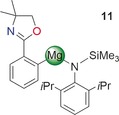
Complex **9** was chosen as an example. Freshly prepared *n*‐butylsodium (168.2 mg, 2.1 mmol) was suspended in methylcyclohexane (15 mL) and then 2,6‐diisopropyl‐*N*‐(trimethylsilyl)aniline (998.0 mg, 4 mmol) was added. The resulting beige suspension was stirred for 1 h and then commercial *n*Bu_2_Mg (2.1 mL, 1 m solution in *n*‐heptane, 2.1 mmol) was introduced by syringe. The reaction mixture was stirred for an additional 1 h. At this juncture, 4,4‐dimethyl‐2‐phenyl‐oxazoline (**12 a**; 341.9 μL, 2 mmol) was added by syringe. Next the mixture was heated to reflux for 1.5 h to give a yellow solution, which was allowed to cool down to ambient temperature. After a period of one week, colourless crystals of **11** grew from the reaction mixture. Attempts to analyse the crystalline material by X‐ray diffraction studies were unsuccessful due to the highly disordered nature within the structure of **11**. These were filtered, washed with *n*‐hexane (3×4 mL) and dried under vacuum (unrefined yield: 290 mg, 0.60 mmol, 30 %). The absolute yield was higher since the filtrate contained a mixture of the product **11** and starting material. The reaction was also studied using two and three molar equivalents of complex **9** and one equivalent of the substrate (2 mmol) giving the same compound **11**. The NMR spectra of isolated crystalline **11** are in agreement with a 1:1 ratio of [N(SiMe_3_)(Dipp)]:[2‐C_6_H_4_‐1‐(oxazoline(Me)_2_)] in **11**. ^1^H NMR (400.13 MHz, C_6_D_6_/[D_8_]THF, 25 °C): *δ*=8.24 (ddd, *J*(H,H)=6.8, 1.2, 0.8 Hz, 1 H; *m*‐C*H*‐Ar), 7.99 (dt, *J*(H,H)=7.6, 0.9 Hz, 1 H; *o*‐C*H*‐Ar), 7.42 (td, *J*(H,H)=7.2, 1.2 Hz, 1 H; *p*‐C*H*‐Ar), 7.20 (td, *J*(H,H)=7.5, 1.4 Hz, 1 H; *m*‐C*H*‐Ar), 7.17 (d, 2 H; C_6_D_6_ overlapping, *m*‐C*H*‐Ar, Dipp), 6.97 (t, *J*(H,H)=7.5 Hz, 1 H; C*H*‐Ar, Dipp), 4.30 (br sept, *J*(H,H)=6.7 Hz, 2 H; ‐C*H*(CH_3_)_2_), 3.69 (s, 2 H; ‐C*H*_2_‐), 1.43 (d, *J*(H,H)=6.9 Hz, 6 H; ‐CH(C*H*_3_)_2_), 1.28 (br d, *J*(H,H)=6.5 Hz, 6 H; ‐CH(C*H*_3_)_2_), 0.94 (br s, 6 H; (‐C*H*_3_)_2_), 0.33 ppm (s, 9 H; ‐Si(C*H*_3_)_3_); ^13^C{^1^H} NMR (100.6 MHz, C_6_D_6_/[D_8_]THF, 25 °C): *δ*=178.9 (*C*_q_‐Mg), 176.0 (*C*_q_‐Ar), 152.5 (N‐*C*_q_‐Ar, Dipp), 145.0 (*o*‐*C*_q_‐Ar, Dipp), 140.1 (*m*‐*C*H‐Ar), 135.0 (*C*_q_‐Ar), 131.0 (*p*‐*C*H‐Ar), 125.7 (*o*‐*C*H‐Ar), 125.1 (*m*‐*C*H‐Ar), 123.3 (*m*‐*C*H‐Ar, Dipp), 119.5 (*p*‐*C*H‐Ar, Dipp), 81.3 (‐*C*H_2_‐), 64.5 (*C*_q_), 28.1 (br, (‐*C*H_3_)_2_), 27.3 (‐*C*H(CH_3_)_2_), 25.3 (‐CH(*C*H_3_)_2_), 25.1 (‐CH(*C*H_3_)_2_), 3.6 ppm (‐Si(C*H*_3_)_3_). NMR spectra also revealed small amounts of methylcyclohexane (from crystallisation) as well as minute traces of 4,4‐dimethyl‐2‐phenyl‐oxazoline and [N(H)(SiMe_3_)(Dipp)] as a result of unavoidable hydrolysis. Elemental analysis calcd (%) for C_26_H_38_MgN_2_OSi: C 69.86, H 8.57, N 6.27; found: C 70.38, H 8.49, N, 6.55.

### Application of metallated compounds in organic synthesis by electrophilic addition reaction

**General procedure**: The aryl substrates 4,4‐dimethyl‐2‐phenyl‐oxazoline (**12 a**) and *N*,*N*‐diisopropylbenzamide (**12 b**) were treated with the appropriate metal complex in methylcyclohexane. All reactions were stirred at/for different temperatures/times and substrate:base stoichiometries of 1:1 and 1:2 were probed. Following metallation, the corresponding 2‐monoiodo derivatives **13 a** and **13 b** were obtained by in situ reaction with an iodine solution in tetrahydrofuran (1 m) at −78 °C. The reaction mixture was allowed to warm up to ambient temperature over a period of 16 h. A saturated aqueous NH_4_Cl solution was added, followed by saturated aqueous Na_2_S_2_O_3_ solution. Extraction of the organic crude with ethyl acetate (3×10 mL), then it was washed with brine (10 mL) and dried over anhydrous MgSO_4_. The solvent was removed under reduced pressure and the crude reaction product was dissolved in CDCl_3_. 1,10‐Phenanthroline was added as internal standard, the yields being calculated by ^1^H NMR spectroscopy (see Tables S10 and S11 in the Supporting Information for details).

The aromatic substrate **12 a** or **12 b** (2 mmol) was added to a reaction mixture of the corresponding monometallic complexes **1**, **2**, [Mg{N(SiMe_3_)(Dipp)}(*n*Bu)] (2 mmol) in methylcyclohexane (15 mL) or the mixed‐metal complexes **9**/**10** (2 mmol/4 mmol) in methylcyclohexane (15 mL/30 mL, respectively). All reactions were stirred at/for 25 °C/24 h and 101 °C/1.5 h respectively. The iodination reaction was carried out as outlined above.

**In situ synthesis of [Mg{N(SiMe_3_)(Dipp)}(*n*Bu)]**: In a Schlenk tube, 2,6‐diisopropyl‐*N*‐(trimethylsilyl)aniline (499.0 mg, 2 mmol) was added to a solution of commercial *n*Bu_2_Mg (2.1 mL, 1 m solution in *n*‐heptane, 2.1 mmol) in bulk methylcyclohexane (15 mL). The reaction mixture was stirred for 1 h before the aromatic substrate was introduced.

## Supporting information

As a service to our authors and readers, this journal provides supporting information supplied by the authors. Such materials are peer reviewed and may be re‐organized for online delivery, but are not copy‐edited or typeset. Technical support issues arising from supporting information (other than missing files) should be addressed to the authors.

Supplementary
