# Supplementary material for: Structural Diversity in Alkali Metal and Alkali Metal Magnesiate Chemistry of the Bulky 2,6‐Diisopropyl‐N‐(trimethylsilyl)anilino Ligand
Source: Chemistry. 2016 Aug 30;22(42):14968–78. doi: 10.1002/chem.201602683 (PMC5096043; doi:10.1002/chem.201602683)
Supplement: Supplementary file 1 — Supplementary [file CHEM-22-14968-s001.pdf]

CHEMISTRY
A European Journal

Supporting Information

Structural Diversity in Alkali Metal and Alkali Metal Magnesiate Chemistry of the Bulky 2,6-Diisopropyl-*N*-(trimethylsilyl)anilino Ligand

M. Ángeles Fuentes, Andoni Zabala, Alan R. Kennedy, and Robert E. Mulvey*[a]

chem_201602683_sm_miscellaneous_information.pdf

Supporting Information Contents

EXPERIMENTAL SECTION

Crystal structure determinations…………………………………………………… 3

NMR spectroscopic studies.. 4

DOSY NMR STUDY

Figures S1-S2; Tables S1-S6. Selected DOSY NMR spectroscopy data…… 4

NMR SPECTRA

Figures S3-S6. ^{1}H; ^{13}C{^{1}H}; ^{1}H, ^{1}H-COSY; ^{1}H, ^{13}C-HSQC NMR spectra of [Na{N($SiMe_3$)(Dipp)}]$_\infty$, **1**…………………………………………………………… 9

Figures S7-S10. ^{1}H; ^{13}C{^{1}H}; ^{1}H, ^{1}H-COSY; ^{1}H, ^{13}C-HSQC NMR spectra of [K{N($SiMe_3$)(Dipp)}]$_\infty$, **2**……………………………………………………………... 11

Figures S11-S15. ^{1}H; ^{7}Li; ^{13}C{^{1}H}; ^{1}H, ^{1}H-COSY; ^{1}H, ^{13}C-HSQC NMR spectra of [Li{N($SiMe_3$)(Dipp)}(PMDETA)], **3**……………………………………….. 13

Figures S16-S19. ^{1}H; ^{13}C{^{1}H}; ^{1}H, ^{1}H-COSY; ^{1}H, ^{13}C-HSQC NMR spectra of [Na{N($SiMe_3$)(Dipp)}(PMDETA)], **4**………………………………………………… 15

Figures S20-S23. ^{1}H; ^{13}C{^{1}H}; ^{1}H, ^{1}H-COSY; ^{1}H, ^{13}C-HSQC NMR spectra of [K{N($SiMe_3$)(Dipp)}(PMDETA)], **5**…………………………………………………… 17

Figures S24-S28. ^{1}H; ^{7}Li; ^{13}C{^{1}H}; ^{1}H, ^{1}H-COSY; ^{1}H, ^{13}C-HSQC NMR spectra of [Li{N($SiMe_3$)(Dipp)}(TMEDA)], **6**…………………………………………… 19

Figures S29-S32. ^{1}H; ^{13}C{^{1}H}; ^{1}H, ^{1}H-COSY; ^{1}H, ^{13}C-HSQC NMR spectra of [Na{N($SiMe_3$)(Dipp)}(TMEDA)], **7**…………………………………………….. 22

Figures S33-S36. ^{1}H; ^{13}C{^{1}H}; ^{1}H, ^{1}H-COSY; ^{1}H, ^{13}C-HSQC NMR spectra of [K{N($SiMe_3$)(Dipp)}(TMEDA)], **8**………………………………………………… 24

Figures S37-S40. ^{1}H; ^{13}C{^{1}H}; ^{1}H, ^{1}H-COSY; ^{1}H, ^{13}C-HSQC NMR spectra of [NaMg{N($SiMe_3$)(Dipp)}$_2$(μ-*n*Bu)]$_\infty$, **9**…………………………………………... 26

Figures S41-S44. ^{1}H; ^{13}C{^{1}H}; ^{1}H, ^{1}H-COSY; ^{1}H, ^{13}C-HSQC NMR spectra of [KMg{N($SiMe_3$)(Dipp)}$_2$(μ-*n*Bu)]$_\infty$, **10**…………………………………………… 28

Figures S45-S48. ^{1}H; ^{13}C{^{1}H}; ^{1}H, ^{1}H-COSY; ^{1}H, ^{13}C-HSQC NMR spectra of [Mg{N($SiMe_3$)(Dipp)}{2-C_6H_4-1-(oxazoline(Me)$_2$)}], **11**……............................…. 30

CRYSTALLOGRAPHIC DATA

Tables S7-S9. Selected crystallographic and refinement data and comparison of selected metric data……………………………………………… 32

REACTIVITY DATA

Tables S10-S11. Selected data from reactivity studies of novel complexes with organic substrates…………………………………………………………….. 35

REFERENCES 36

EXPERIMENTAL SECTION

General procedures. All reactions and manipulations were performed under a protective atmosphere of dry pure argon gas using standard Schlenk tube or glovebox techniques. NMR spectra were recorded on a Bruker DPX 400 NMR spectrometer, operating at 400.13 MHz for ^{1}H, 155.5 MHz for ^{7}Li and 100.6 MHz for ^{13}C. ^{1}H and ^{13}C{^{1}H} spectra were referenced to the appropriate solvent signal, ^{7}Li NMR spectra were referenced against LiCl in D_2O at 0.00 ppm. The following abbreviations are used: Dipp = 2,6-*i*Pr_2-C_6H_3, PMDETA = *N*,*N*,*N′*,*N″*,*N″*-pentamethyldiethylenetriamine and TMEDA = *N*,*N*,*N′*,*N′*-tetramethylethylenediamine; TMS = tetramethylsilane.

Crystal Structure Determinations. Single-crystal data were measured at 123(2) K on Oxford Diffraction Diffractometers with Mo-*Kα* radiation (λ = 0.71073 Å) for **1** – **6** and **8** – **10**. Measurements were at 230(2) K with Cu-*Kα* radiation (λ = 1.5418 Å) for **7**. The structures were refined to convergence on F^2 and against all independent reflections by full-matrix least-squares using SHELXL programs.[1] For **1**, the equivalent of 41 electrons were removed from approx 414 Å^3 of void space using the program SQUEEZE.[2] This electron density was believed to be due to disordered and partially present solvent molecules. The TMEDA ligand (except for nitrogen atoms) and an *i*Pr unit of **6** were modelled as disordered over two sites. Restraints were applied to N-C and C-C distances and to displacement ellipsoid in order to ensure normal behaviour. A single TMEDA ligand of **8** and the solvent of crystallisation (methylcyclohexane) and one component of a -$SiMe_3$ group of **10** were treated in similar fashion as disordered over two sites. CCDC-1482316 (**1**), CCDC-1482317 (**2**), CCDC-1482318 (**3**), CCDC-1482319 (**4**), CCDC-1482320 (**5**), CCDC-1482321 (**6**), CCDC-1482322 (**7**), CCDC-1482323 (**8**), CCDC-1482324 (**9**) and CCDC-1482325 (**10**) contain the supplementary crystallographic data for this paper. These data can be obtained free of charge from the Cambridge Crystallographic Data Centre via www.ccdc.cam.ac.uk/data_request/cif.

NMR spectroscopic studies

^{1}H DOSY-NMR Analysis details. Diffusion-Ordered SpectroscopY (DOSY) NMR experiments were performed on a Bruker AVANCE 400 MHz NMR spectrometer at 25ºC operating at 400.13 MHz for ^{1}H under TopSpin (version 2.0, Bruker Biospin, Karlsruhe) and equipped with a BBFO-*z*-atm probe with actively shielded *z*-gradient coil capable of delivering a maximum gradient strength of 54 G cm^{-1}. Diffusion-ordered NMR data were acquired using the Bruker pulse program *dstegp3s* with a double stimulated echo with three spoiling gradients. Sine-shaped gradient pulses were used with a duration of 4 ms together with a diffusion period of 100 ms. Gradient recovery delays of 200 μs followed the application of each gradient pulse. Data were systematically accumulated by linearly varying the diffusion encoding gradients over a range from 2% to 95% of maximum for 64 gradient increment values. The signal decay dimension on the *pseudo*-2D data was generated by Fourier transformation of the time-domain data. DOSY plot were generated by use of the DOSY processing module of TopSpin. Parameters were optimized empirically to find the best quality of data for presentation purposes. Diffusion coefficients (D) were calculated by fitting intensity data to the Stejskal-Tanner expression. The external standard was tetramethylsilane, and we used the external calibration curves method recently published by Stalke.[3] The molecular weight (MW) was estimated by DOSY-NMR using the diffusion coefficients for the signals corresponding to the species present in solution.

DOSY NMR STUDY

^{1}H DOSY-NMR Study of $[NaMg\{N(SiMe_3)(Dipp)\}_2(\mu\text{-}n\mathrm{Bu})]_\infty$, 9 in $[D_8]$THF solution

Following the literature procedure,[3a] a sample (20 mmol/L) was prepared using a 1:1 mixture of crystalline complex **9** and TMS as an external standard which was dissolved in the appropriate deuterated solvent ($[D_8]$THF). The MW(DOSY) of species in solution were estimated by using the D values for the complex **9** in deuterated solvent solution (Figure S1, Tables S2-S3).

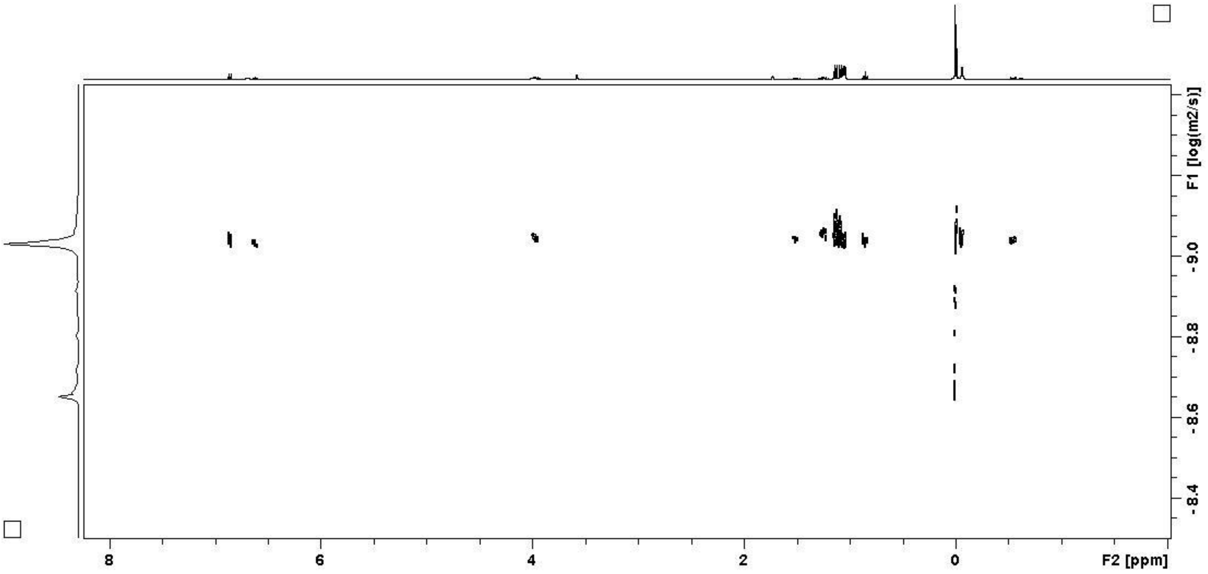

Figure S1. ^{1}H DOSY-NMR plot of complex **9** in solution (400.13 MHz, $[D_8]THF$, 25ºC).

**Table S1.** External calibration curves recently published by Stalke.[3]				
**Solvent**	**Internal reference**	**D (m^2 s^{-1})**	**logD, fix**	**Calibration curve**
$[D_8]THF$	TMS	2.044E-9	-8.7018	Dissipated Spheres + Ellipsoids (in THF)

Studies in solution state reveal the cleavage of the complex **9** into two separate species corresponding to the monometallic components, (Tables S2-S3).

**Table S2.** Diffusion coefficients and corresponding calculated MW(DOSY) for components present in $[D_8]THF$ solution.			
**Component of spectrum**	**D (m^2 s^{-1})**	**logD, norm**	**MW(DOSY)[a] (g mol^{-1})**
$[([D_8]THF)Mg\{N(SiMe_3)(Dipp)\}$ (μ-*n*Bu)]	9.336E-10	-9.0421	386
$[([D_8]THF)_2Na\{N(SiMe_3)(Dipp)\}]$	9.149E-10	-9.0509	399
[a] MW using the external calibration curves recently published by Stalke.[3]			

The MW(DOSY) values obtained were compared to the theoretical MW of various species which may be present in the solution. The error of the MW(DOSY) values with respect to these species was also determined. Results are shown in Table S3.

Table S3. Diffusion coefficients and corresponding calculated MW(DOSY) for components present in [D_8]THF solution.

**Possible species**	**MW[a] (g mol^{-1})**	**MW(DOSY)[b] (g mol^{-1})**	**Error (%) in MW(DOSY)**
[([D_8]THF)Mg{N($SiMe_3$)(Dipp)}(*μ*-*n*Bu)]	409	386	-6
[([D_8]THF)$_2$Mg{N($SiMe_3$)(Dipp)}(*μ*-*n*Bu)]	490	386	-21
[([D_8]THF)$_2$Na{N($SiMe_3$)(Dipp)}]	431	399	-7

[a] Theoretical MW. [b] MW using the external calibration curves recently published by Stalke.[3]

As shown in Figure S1 and Tables S2-S3, the data could be consistent with [([D_8]THF)Mg{N($SiMe_3$)(Dipp)}(*μ*-*n*Bu)] and [([D_8]THF)$_2$Na{N($SiMe_3$)(Dipp)}] existing in [D_8]THF solution.

^{1}H DOSY-NMR Study of [KMg{N($SiMe_3$)(Dipp)}$_2$(*μ*-*n*Bu)]$_\infty$, 10 in [D_8]THF solution

According to the literature,[3a] a sample (20 mmol/L) was prepared using a 1:1 mixture of crystalline complex **10** and TMS as an external standard which were dissolved in the appropriate deuterated solvent ([D_8]THF). The MW(DOSY) of species in solution were estimated by using the D values for the complex **10** in deuterated solvent solution (Figure S2, Tables S5-S6).

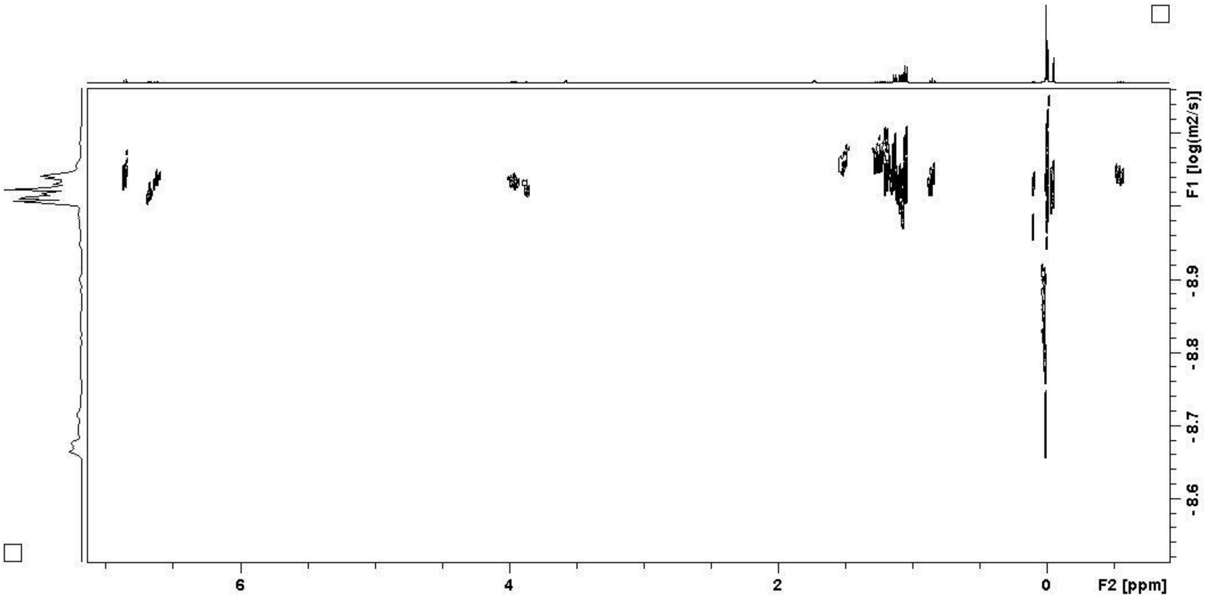

Figure S2. ^{1}H DOSY-NMR plot of complex **10** in solution (400.13 MHz, [D_8]THF, 25ºC).

**Table S4.** External calibration curves recently published by Stalke.[3]				
**Solvent**	**Internal reference**	**D (m^2 s^{-1})**	**logD, fix**	**Calibration curve**
[D_8]THF	TMS	2.044E-9	-8.7018	Dissipated Spheres + Ellipsoids (in THF)

Studies in solution state reveal the cleavage of the complex **10** into two separate species corresponding to the monometallic components, (Tables S5-S6).

**Table S5.** Diffusion coefficients and corresponding calculated MW(DOSY) for components present in [D_8]THF solution.			
**Component of spectrum**	**D (m^2 s^{-1})**	**logD, norm**	**MW(DOSY)[a] (g mol^{-1})**
[([D_8]THF)Mg{N($SiMe_3$)(Dipp)}(*μ*-*n*Bu)]	9.221E-10	-9.0475	394
[([D_8]THF)K{N($SiMe_3$)(Dipp)}]	9.544E-10	-9.0326	371
[a] MW using the external calibration curves recently published by Stalke.[3]			

The MW(DOSY) values obtained were compared to the theoretical MW of various species which may be present in the solution. The error of the MW(DOSY) values with respect to these species was also determined. The results are shown in Table S6.

Table S6. Diffusion coefficients and corresponding calculated MW(DOSY) for components present in [D_8]THF solution.

Possible species	MW[a] (g mol^{-1})	MW(DOSY)[b] (g mol^{-1})	Error (%) in MW(DOSY)
[([D_8]THF)Mg{N($SiMe_3$)(Dipp)}(*μ*-*n*Bu)]	409	394	-4
[([D_8]THF)$_2$Mg{N($SiMe_3$)(Dipp)}(*μ*-*n*Bu)]	490	394	-20
[([D_8]THF)K{N($SiMe_3$)(Dipp)}]	367	371	+1
[([D_8]THF)$_2$K{N($SiMe_3$)(Dipp)}]	447	371	-17

[a] Theoretical MW. [b] MW using the external calibration curves recently published by Stalke.[3]

As shown in Figure S1 and Tables S5-S6, the data could be consistent with [([D_8]THF)Mg{N($SiMe_3$)(Dipp)}(*μ*-*n*Bu)] and [([D_8]THF)K{N($SiMe_3$)(Dipp)}] existing in [D_8]THF solution.

NMR SPECTRA

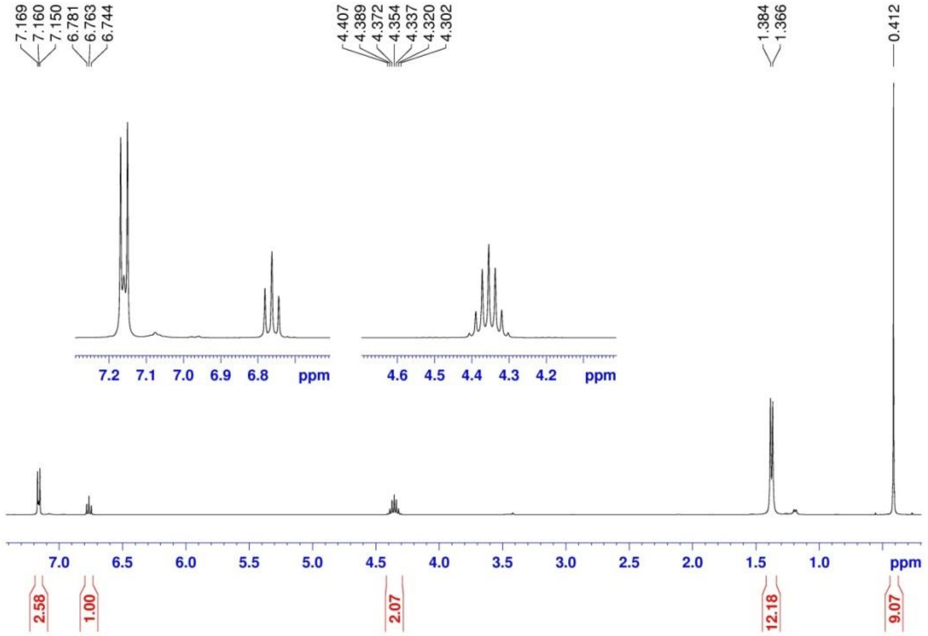

Figure S3. ^{1}H NMR spectrum of [Na{N(SiMe$_3$)(Dipp)}]$_\infty$, **1** (400.13 MHz, C_6D_6/[D_8]THF, 25ºC).

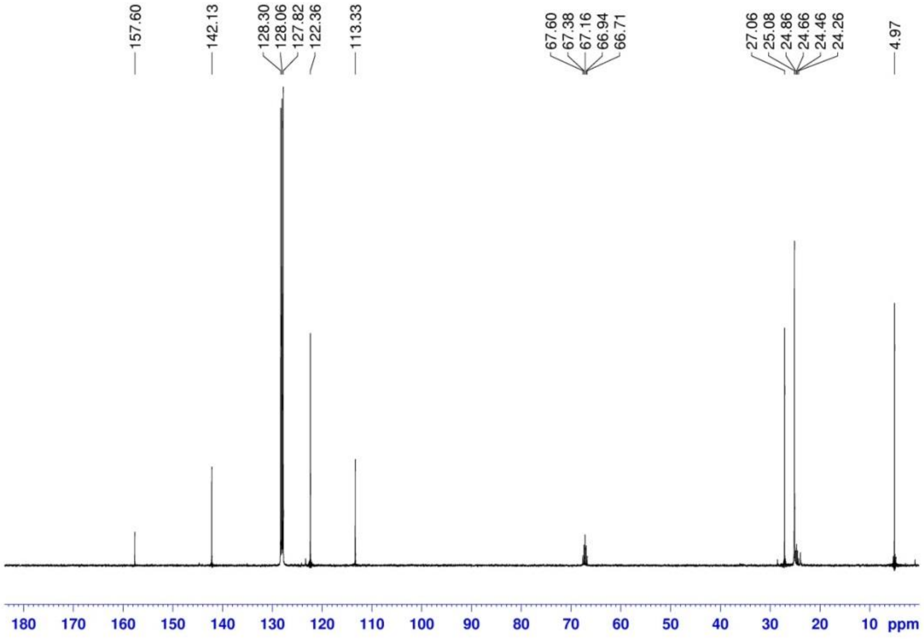

Figure S4. $^{13}C\{^1H\}$ NMR spectrum of [Na{N(SiMe$_3$)(Dipp)}]$_\infty$, **1** (100.6 MHz, C_6D_6/[D_8]THF, 25ºC).

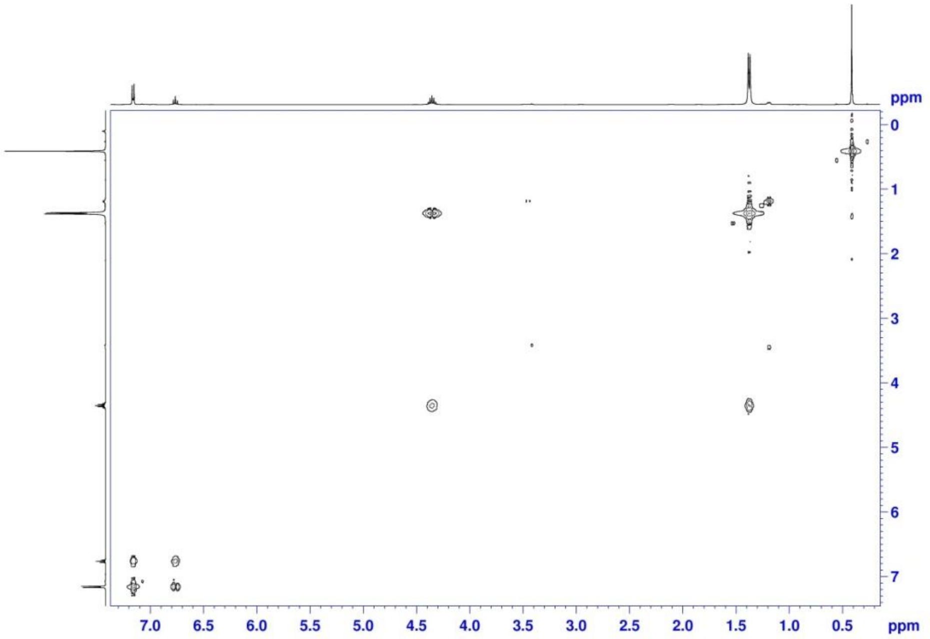

Figure S5. ^{1}H, ^{1}H-COSY NMR spectrum of [Na{N(SiMe$_3$)(Dipp)}]$_\infty$, **1** (400.13 MHz, C_6D_6/[D_8]THF, 25ºC).

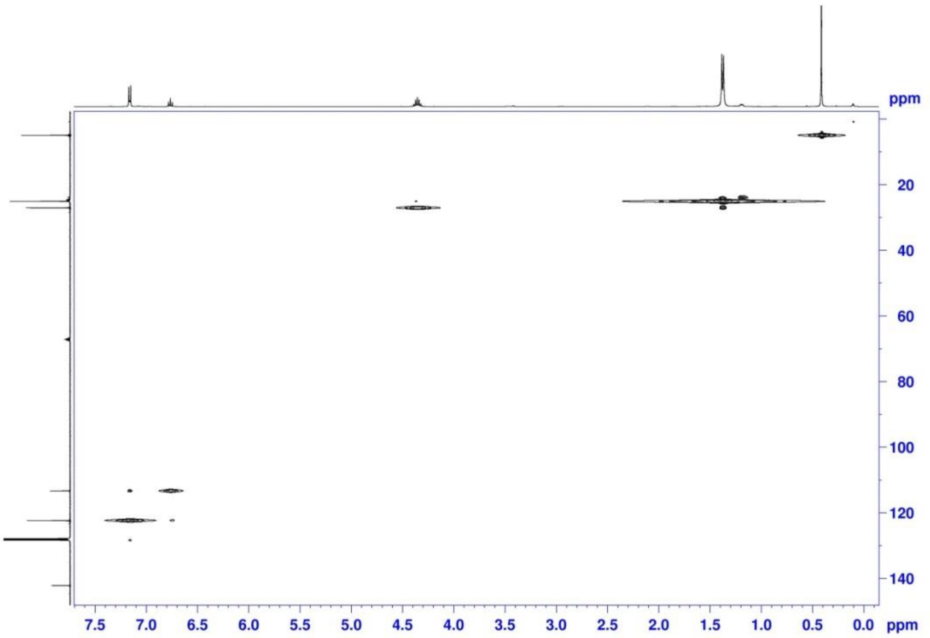

Figure S6. ^{1}H, ^{13}C-HSQC NMR spectrum of [Na{N(SiMe$_3$)(Dipp)}]$_\infty$, **1** (400.13 MHz, C_6D_6/[D_8]THF, 25ºC).

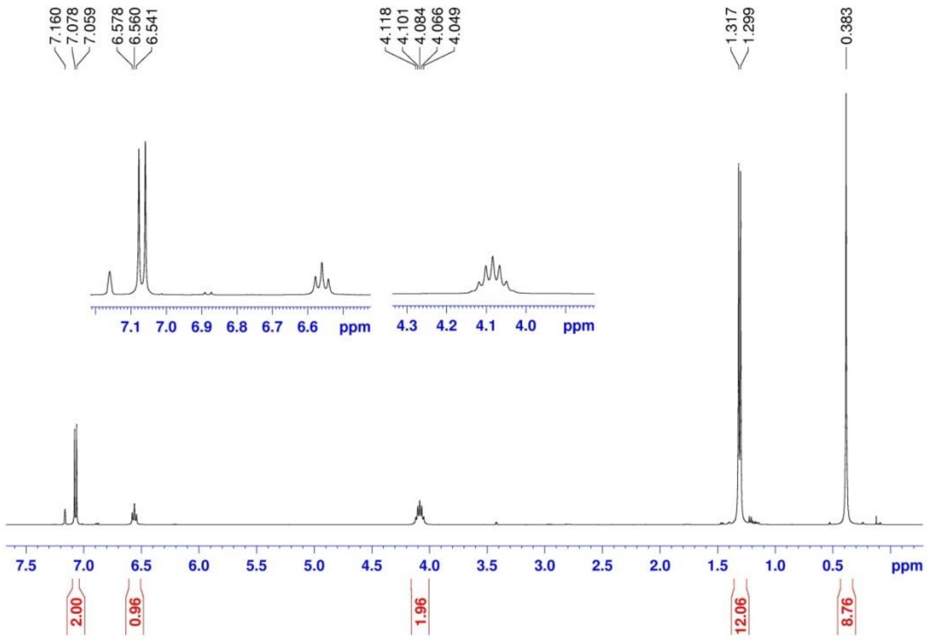

Figure S7. ^{1}H NMR spectrum of [K{N(SiMe$_3$)(Dipp)}]$_\infty$, **2** (400.13 MHz, C_6D_6/[D$_8$]THF, 25°C).

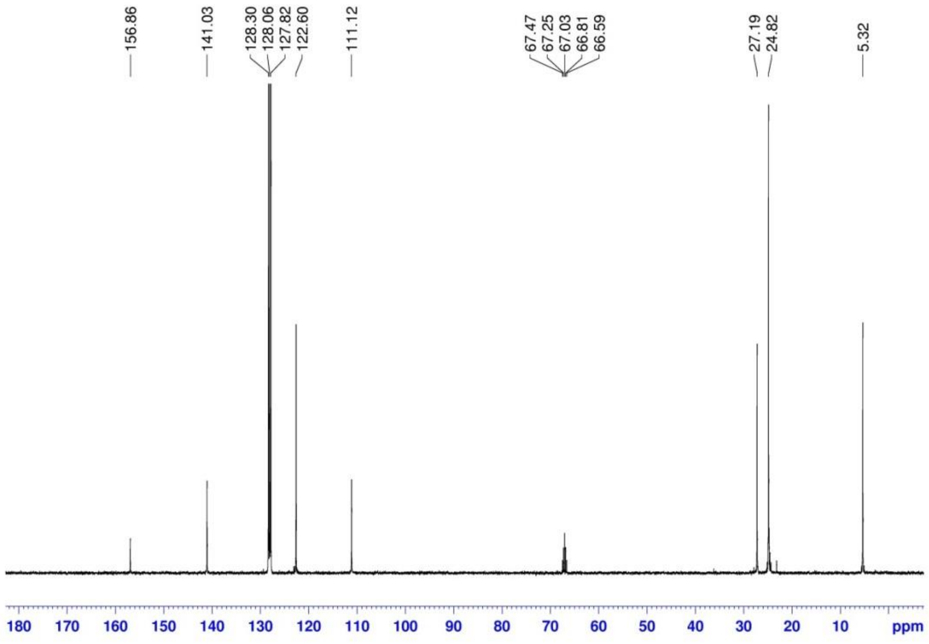

Figure S8. $^{13}C\{^{1}H\}$ NMR spectrum of [K{N(SiMe$_3$)(Dipp)}]$_\infty$, **2** (100.6 MHz, C_6D_6/[D$_8$]THF, 25°C).

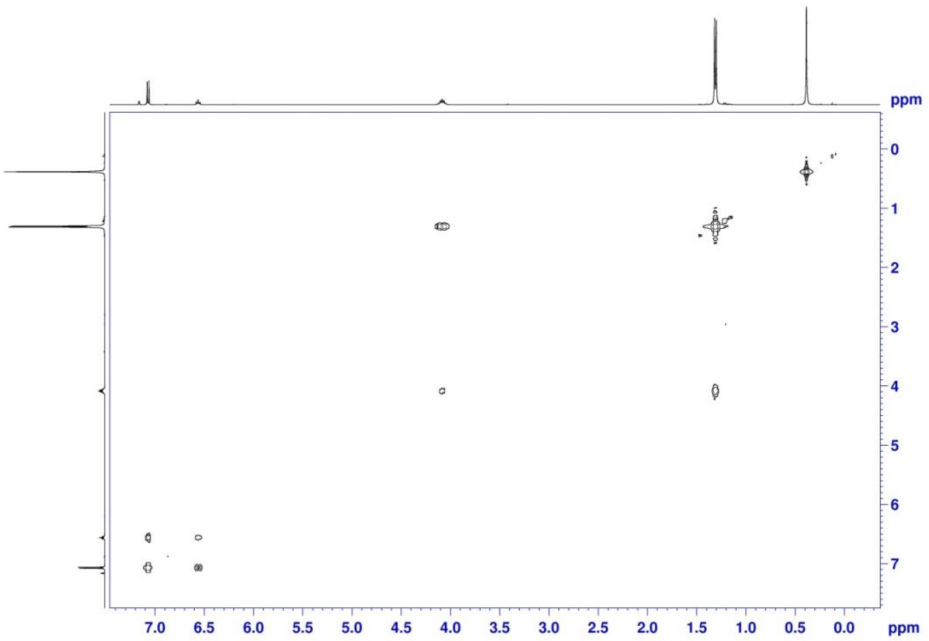

Figure S9. ^{1}H, ^{1}H-COSY NMR spectrum of [K{N($SiMe_3$)(Dipp)}]$_\infty$, **2** (400.13 MHz, C_6D_6/[D_8]THF, 25ºC).

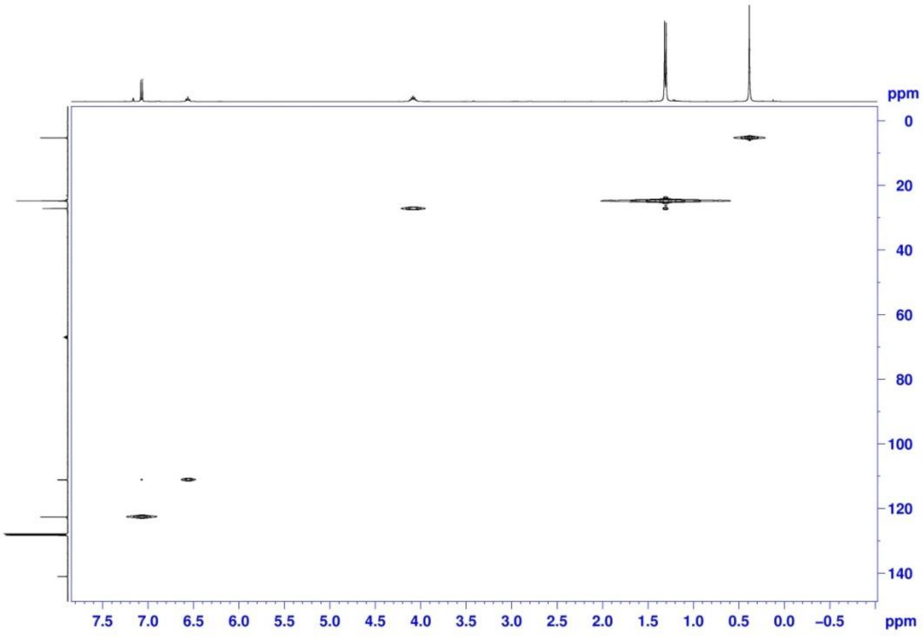

Figure S10. ^{1}H, ^{13}C-HSQC NMR spectrum of [K{N($SiMe_3$)(Dipp)}]$_\infty$, **2** (400.13 MHz, C_6D_6/[D_8]THF, 25ºC).

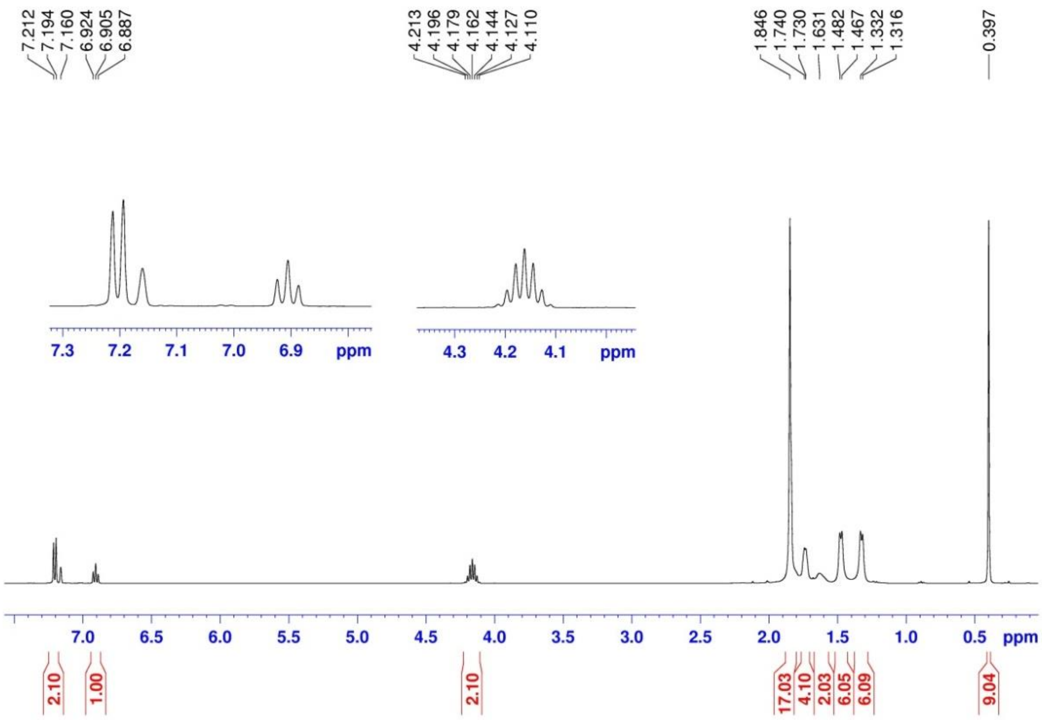

Figure S11. ^{1}H NMR spectrum of [Li{N($SiMe_3$)(Dipp)}(PMDETA)], **3** (400.13 MHz, C_6D_6, 25ºC).

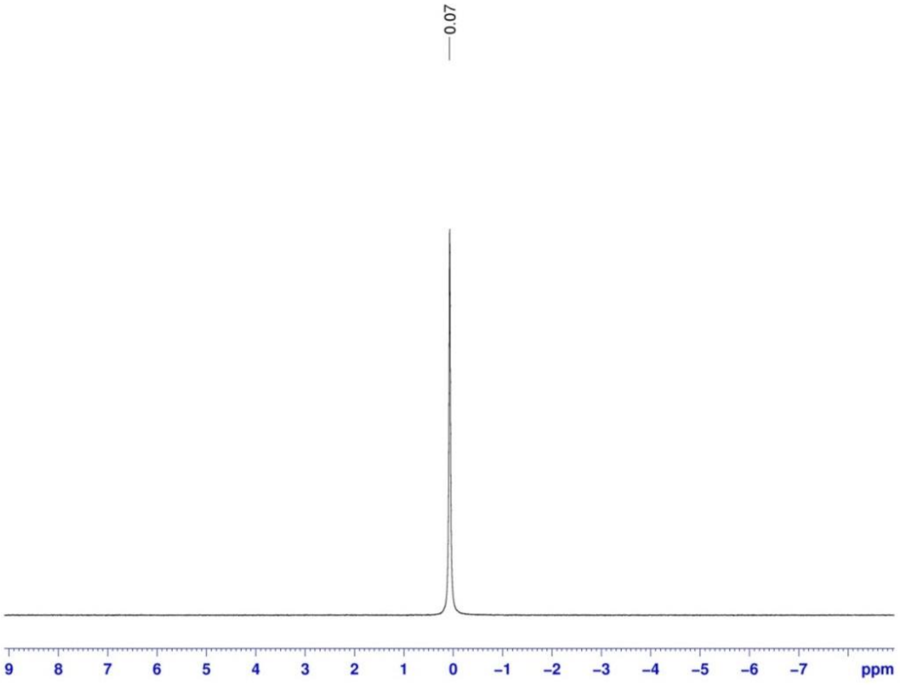

Figure S12. ^{7}Li NMR spectrum of [Li{N($SiMe_3$)(Dipp)}(PMDETA)], **3** (155.5 MHz, C_6D_6, 25ºC).

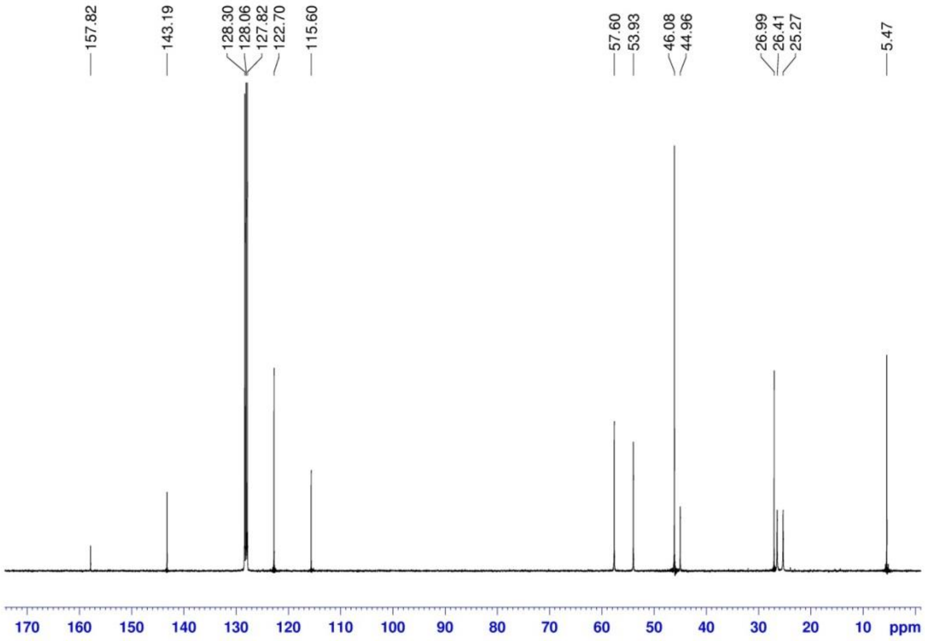

Figure S13. $^{13}C\{^1H\}$ NMR spectrum of $[Li\{N(SiMe_3)(Dipp)\}(PMDETA)]$, **3** (100.6 MHz, C_6D_6, 25ºC).

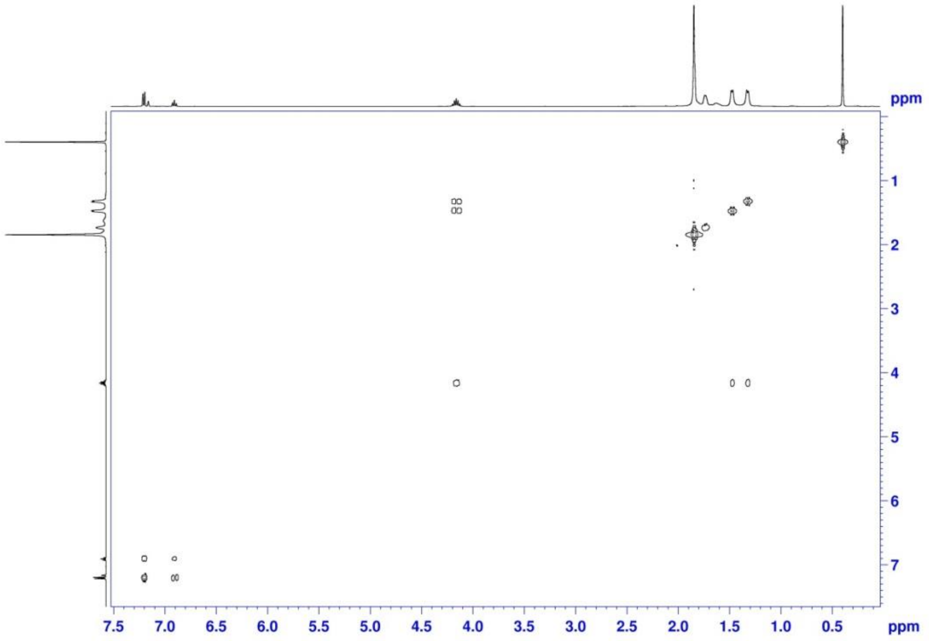

Figure S14. ^1H, ^1H-COSY NMR spectrum of $[Li\{N(SiMe_3)(Dipp)\}(PMDETA)]$, **3** (400.13 MHz, C_6D_6, 25ºC).

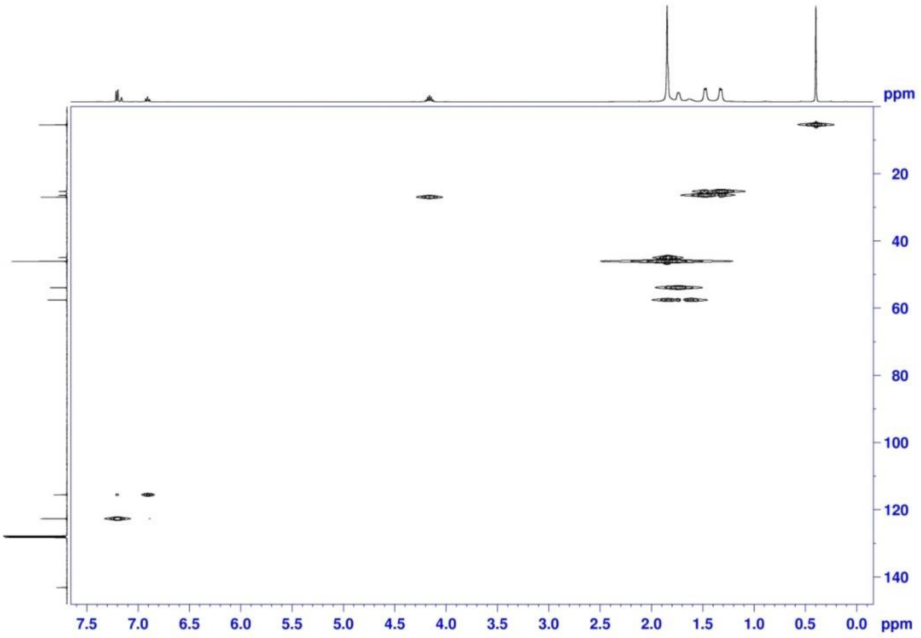

Figure S15. ^{1}H, ^{13}C-HSQC NMR spectrum of [Li{N($SiMe_3$)(Dipp)}(PMDETA)], **3** (400.13 MHz, C_6D_6, 25ºC).

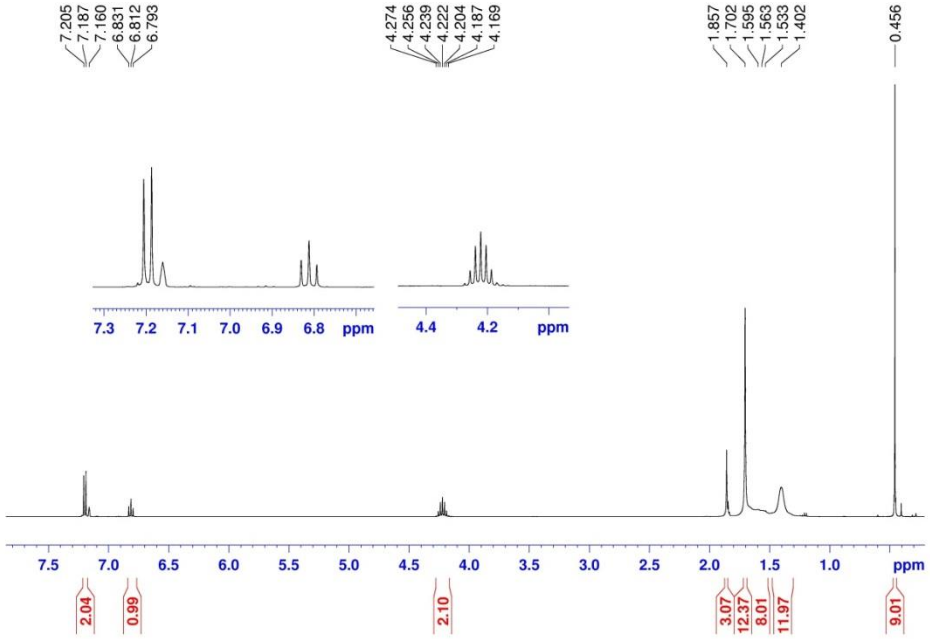

Figure S16. ^{1}H NMR spectrum of [Na{N($SiMe_3$)(Dipp)}(PMDETA)], **4** (400.13 MHz, C_6D_6, 25ºC).

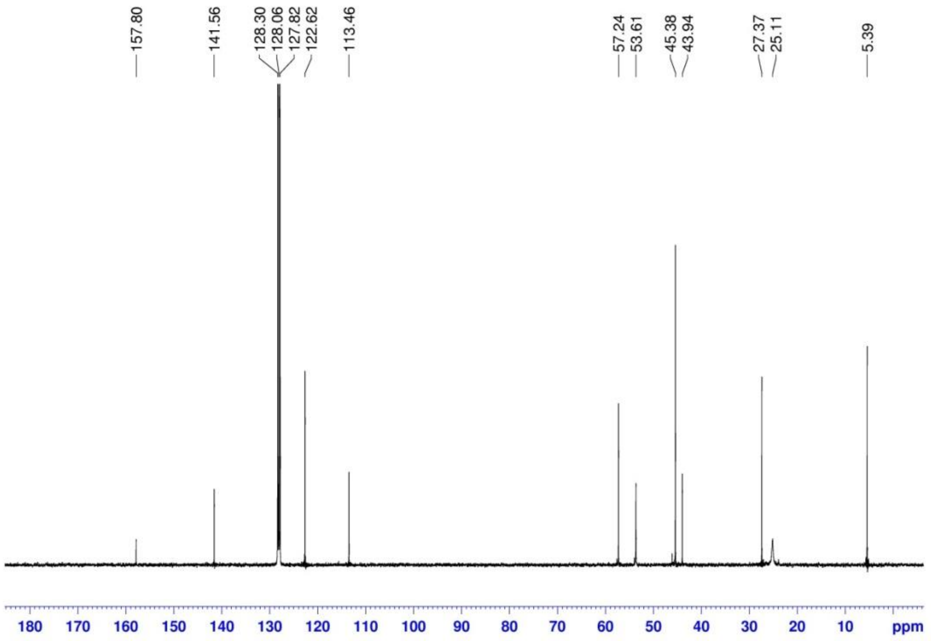

Figure S17. $^{13}C\{^{1}H\}$ NMR spectrum of [Na{N(SiMe$_3$)(Dipp)}(PMDETA)], **4** (100.6 MHz, C_6D_6, 25ºC).

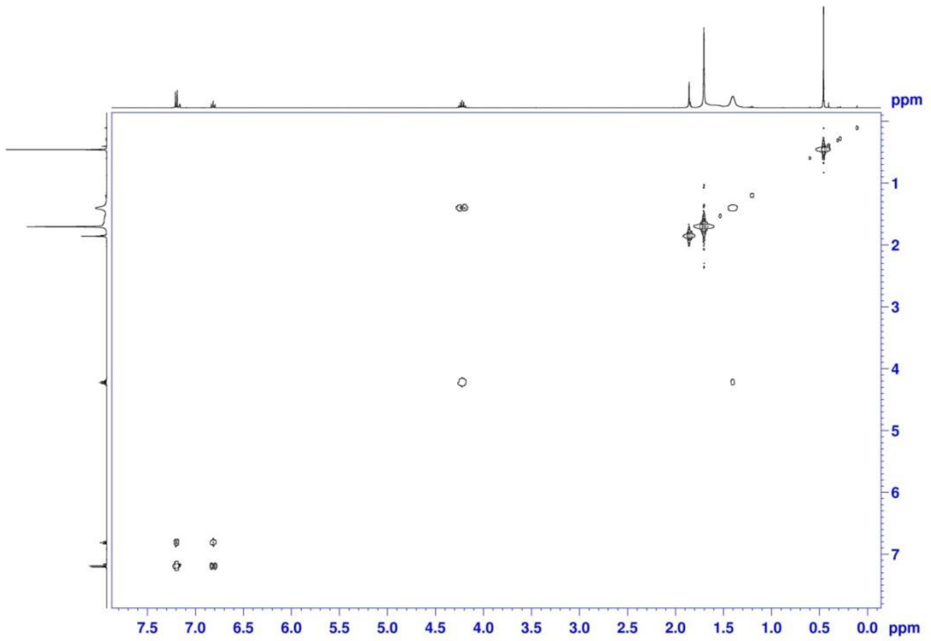

Figure S18. ^{1}H, ^{1}H-COSY NMR spectrum of [Na{N(SiMe$_3$)(Dipp)}(PMDETA)], **4** (400.13 MHz, C_6D_6, 25ºC).

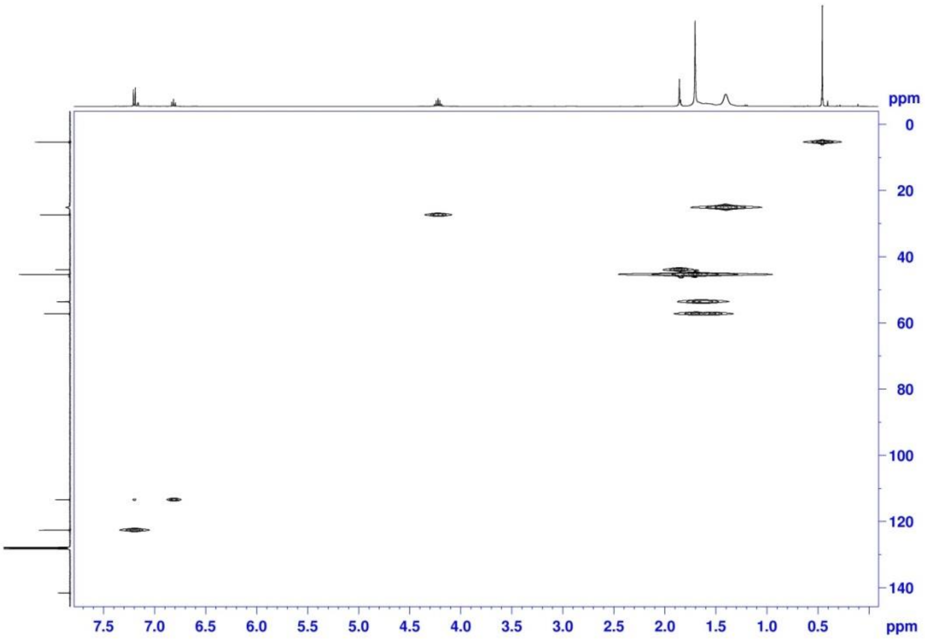

Figure S19. ^{1}H, ^{13}C-HSQC NMR spectrum of [Na{N($SiMe_3$)(Dipp)}(PMDETA)], **4** (400.13 MHz, C_6D_6, 25ºC).

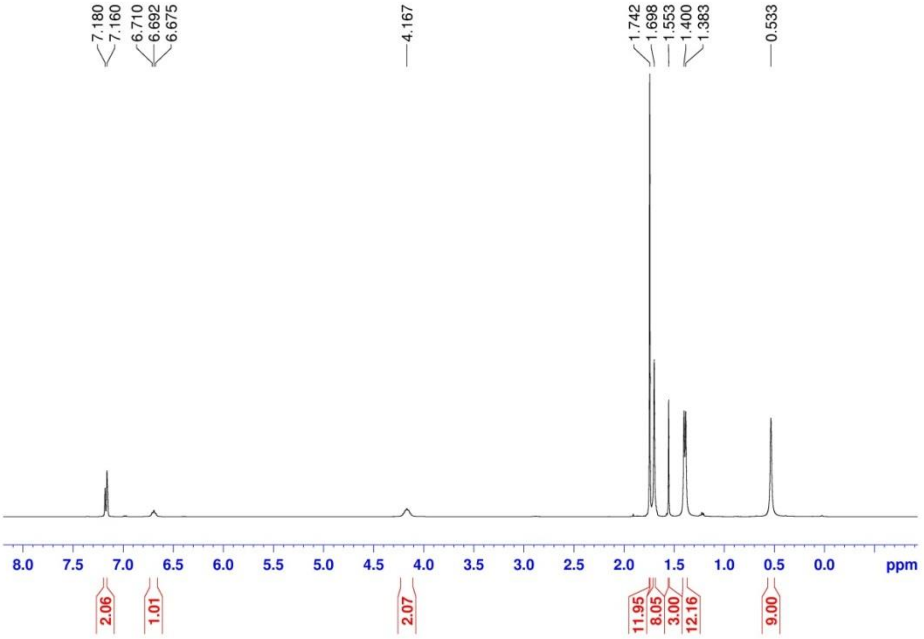

Figure S20. ^{1}H NMR spectrum of [K{N($SiMe_3$)(Dipp)}(PMDETA)]$_2$, **5** (400.13 MHz, C_6D_6, 25ºC).

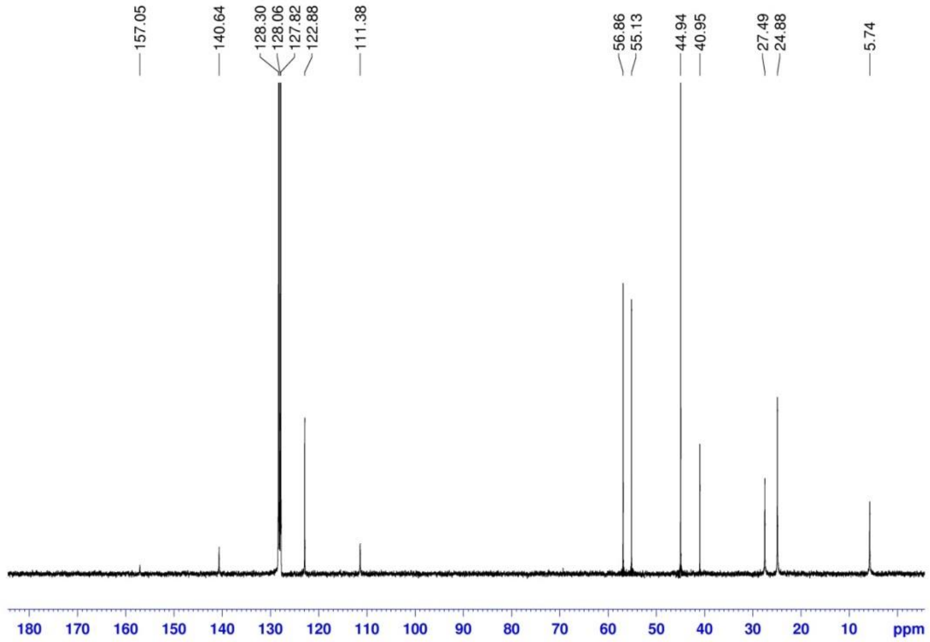

Figure S21. $^{13}C\{^{1}H\}$ NMR spectrum of $[K\{N(SiMe_3)(Dipp)\}(PMDETA)]_2$, **5** (100.6 MHz, C_6D_6, 25ºC).

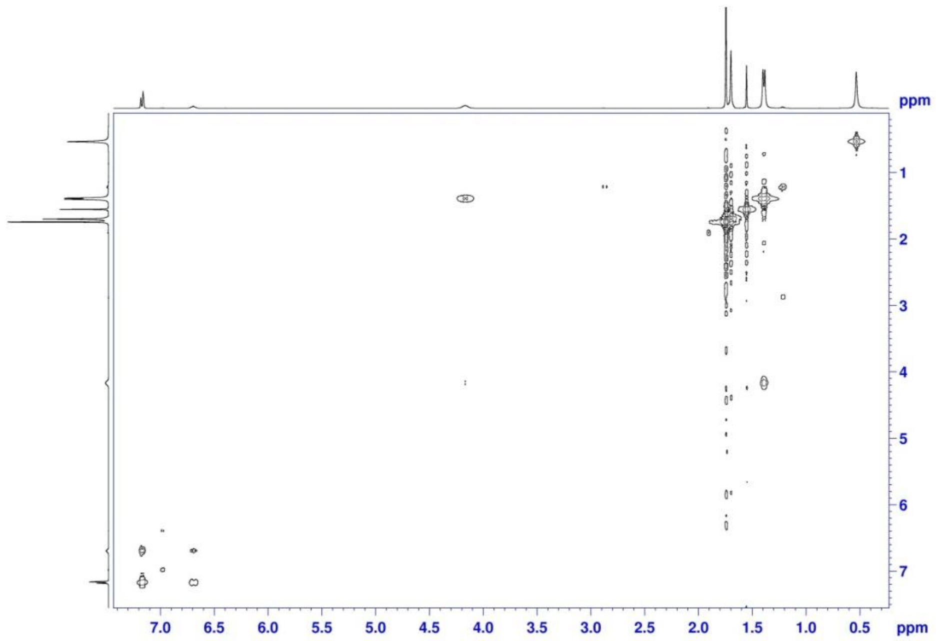

Figure S22. ^{1}H, ^{1}H-COSY NMR spectrum of $[K\{N(SiMe_3)(Dipp)\}(PMDETA)]_2$, **5** (400.13 MHz, C_6D_6, 25ºC).

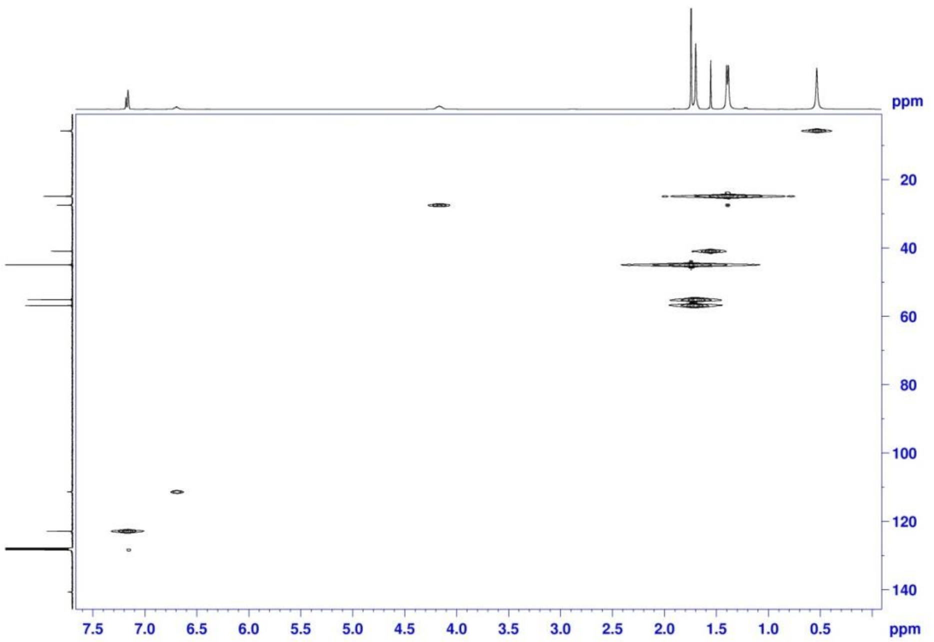

Figure S23. ^{1}H, ^{13}C-HSQC NMR spectrum of [K{N($SiMe_3$)(Dipp)}(PMDETA)]$_2$, **5** (400.13 MHz, C_6D_6, 25ºC).

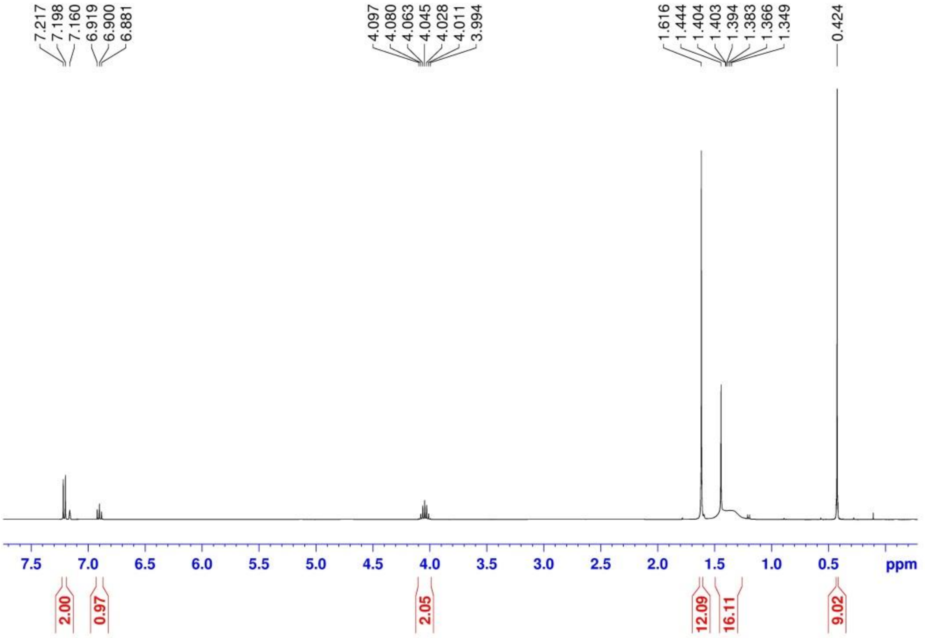

Figure S24. ^{1}H NMR spectrum of [Li{N($SiMe_3$)(Dipp)}(TMEDA)], **6** (400.13 MHz, C_6D_6, 25ºC).

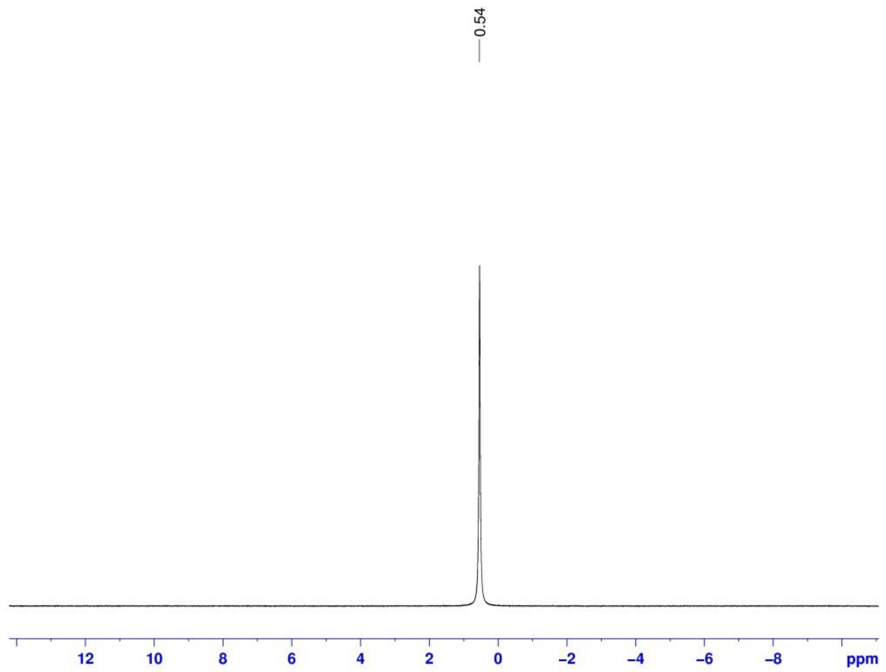

Figure S25. ^{7}Li NMR spectrum of [Li{N($SiMe_3$)(Dipp)}(TMEDA)], **6** (155.5 MHz, C_6D_6, 25ºC).

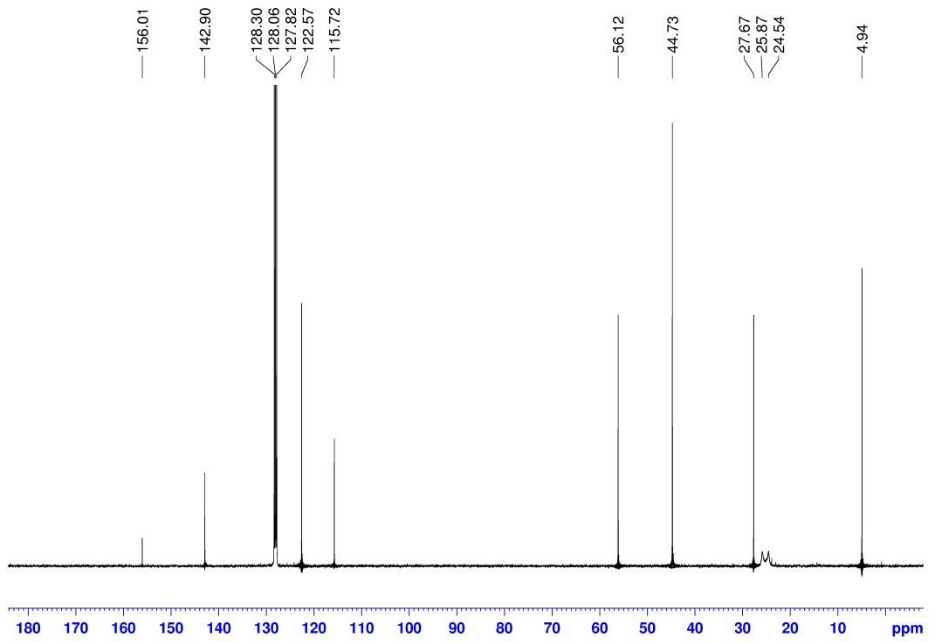

Figure S26. $^{13}C\{^{1}H\}$ NMR spectrum of [Li{N($SiMe_3$)(Dipp)}(TMEDA)], **6** (100.6 MHz, C_6D_6, 25ºC).

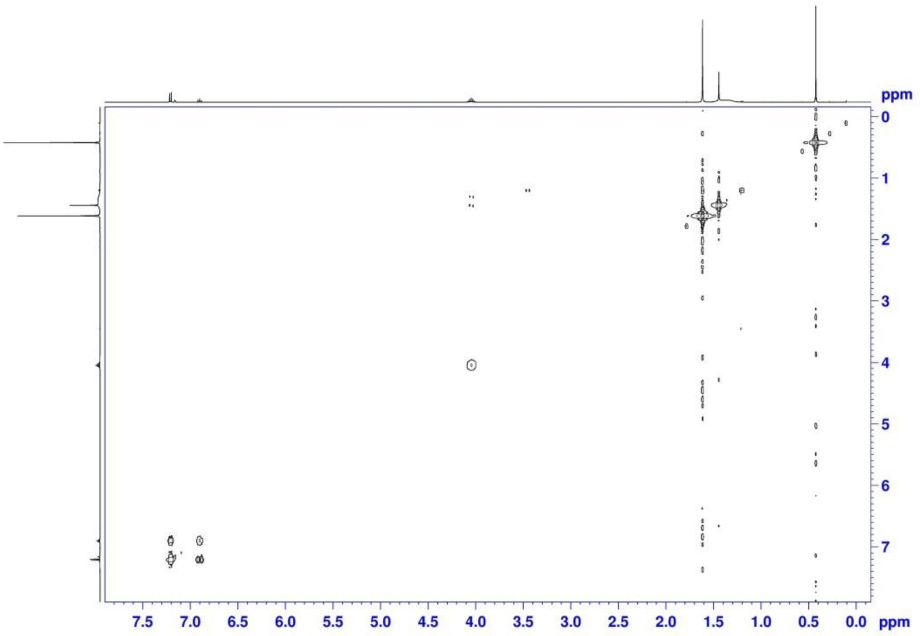

Figure S27. ^{1}H, ^{1}H-COSY NMR spectrum of [Li{N($SiMe_3$)(Dipp)}(TMEDA)], **6** (400.13 MHz, C_6D_6, 25ºC).

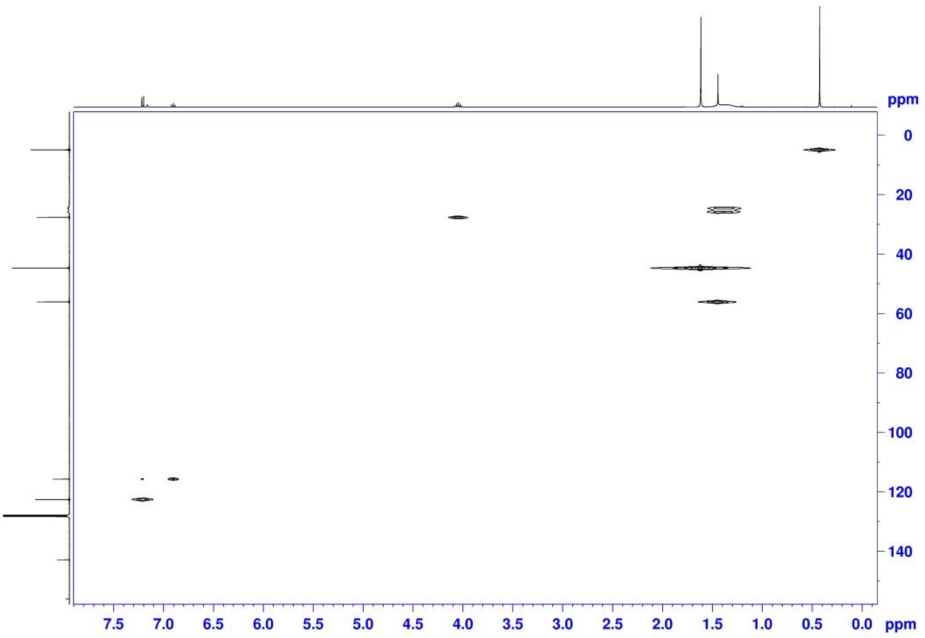

Figure S28. ^{1}H, ^{13}C-HSQC NMR spectrum of [Li{N($SiMe_3$)(Dipp)}(TMEDA)], **6** (400.13 MHz, C_6D_6, 25ºC).

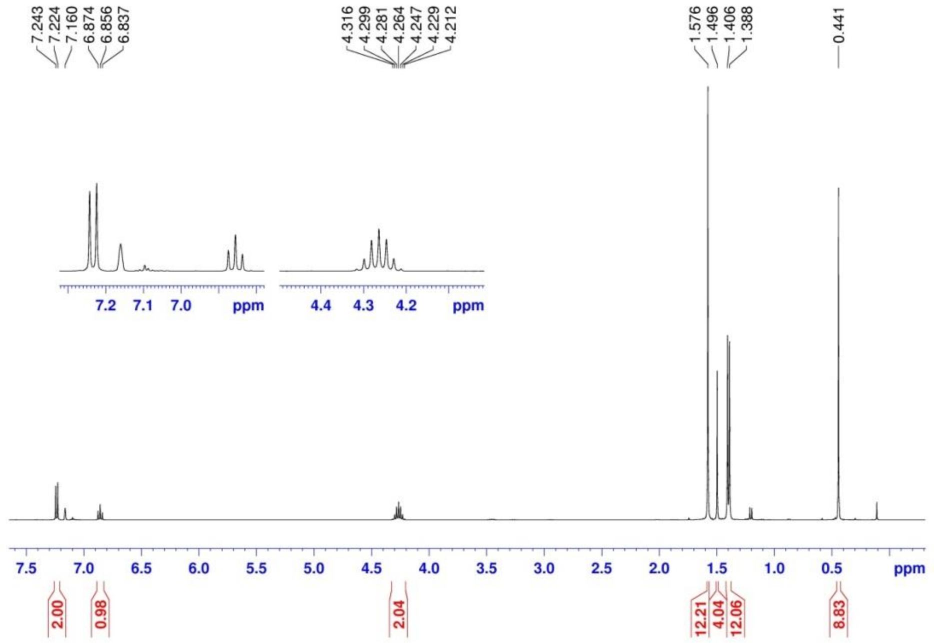

Figure S29. ^{1}H NMR spectrum of [Na{N($SiMe_3$)(Dipp)}(TMEDA)]$_2$, **7** (400.13 MHz, C_6D_6, 25ºC).

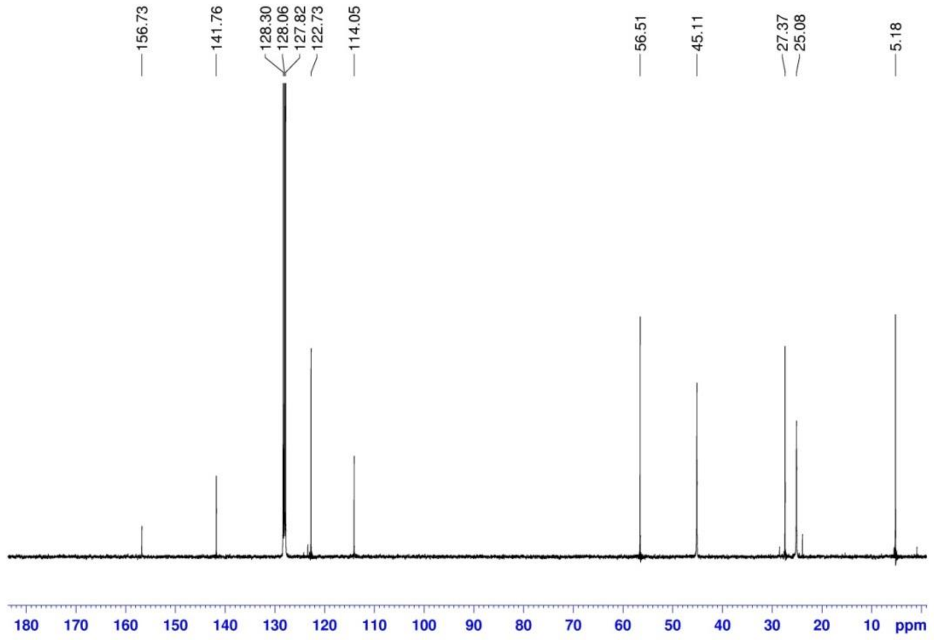

Figure S30. ^{13}C{^{1}H} NMR spectrum of [Na{N($SiMe_3$)(Dipp)}(TMEDA)]$_2$, **7** (100.6 MHz, C_6D_6, 25ºC).

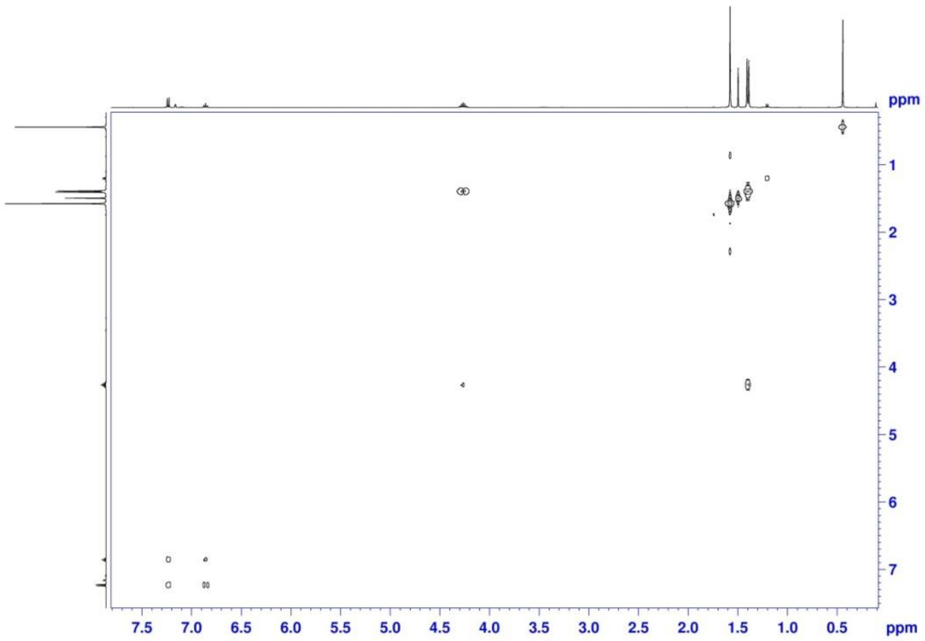

Figure S31. ^{1}H, ^{1}H-COSY NMR spectrum of $[Na\{N(SiMe_3)(Dipp)\}(TMEDA)]_2$, **7** (400.13 MHz, C_6D_6, 25ºC).

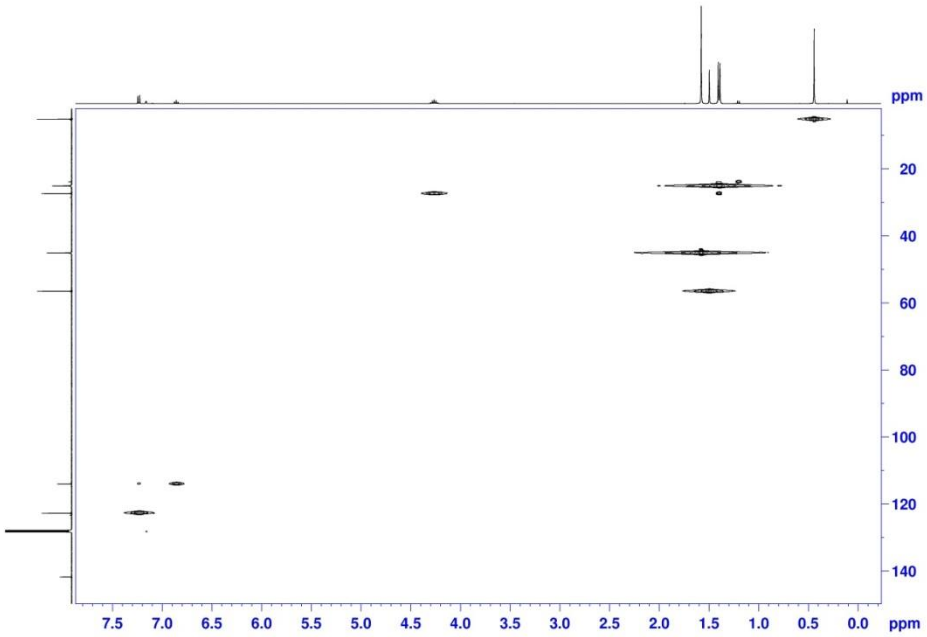

Figure S32. ^{1}H, ^{13}C-HSQC NMR spectrum of $[Na\{N(SiMe_3)(Dipp)\}(TMEDA)]_2$, **7** (400.13 MHz, C_6D_6, 25ºC).

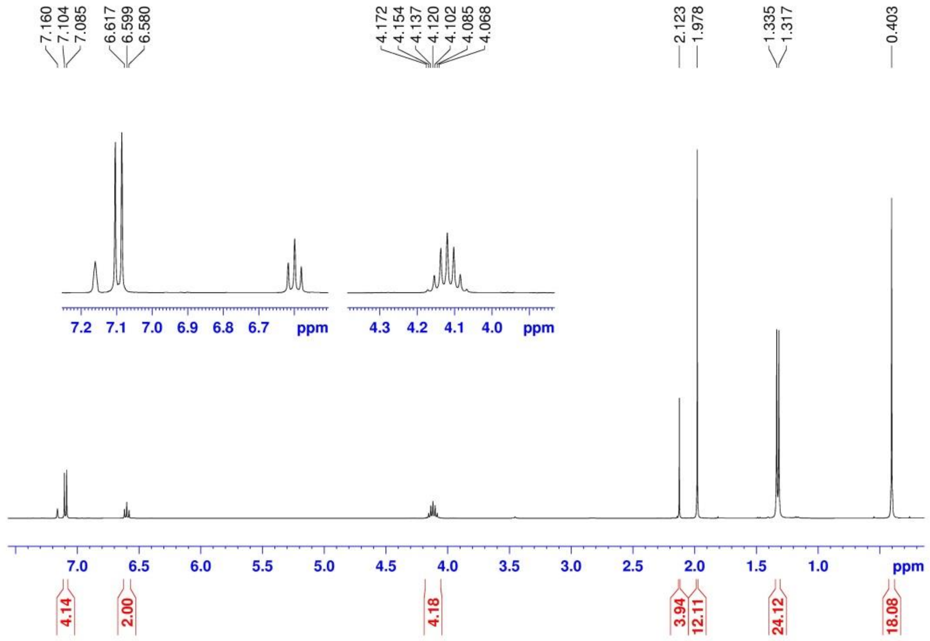

Figure S33. ^{1}H NMR spectrum of [{K{N(SiMe$_3$)(Dipp)}}$_2$(TMEDA)]$_\infty$, **8** (400.13 MHz, C_6D_6/[D$_8$]THF, 25ºC).

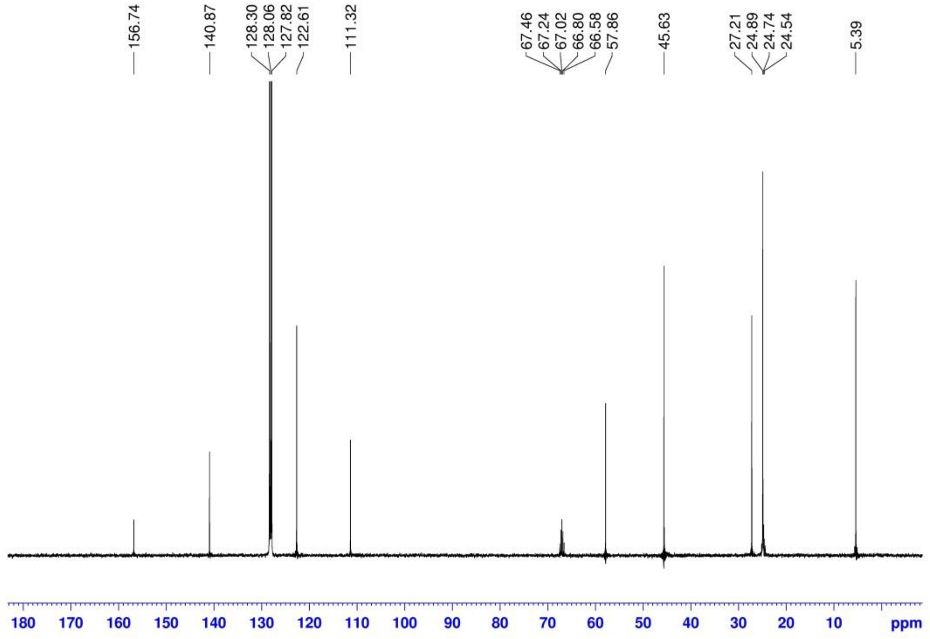

Figure S34. ^{13}C{^{1}H} NMR spectrum of [{K{N(SiMe$_3$)(Dipp)}}$_2$(TMEDA)]$_\infty$, **8** (100.6 MHz, C_6D_6/[D$_8$]THF, 25ºC).

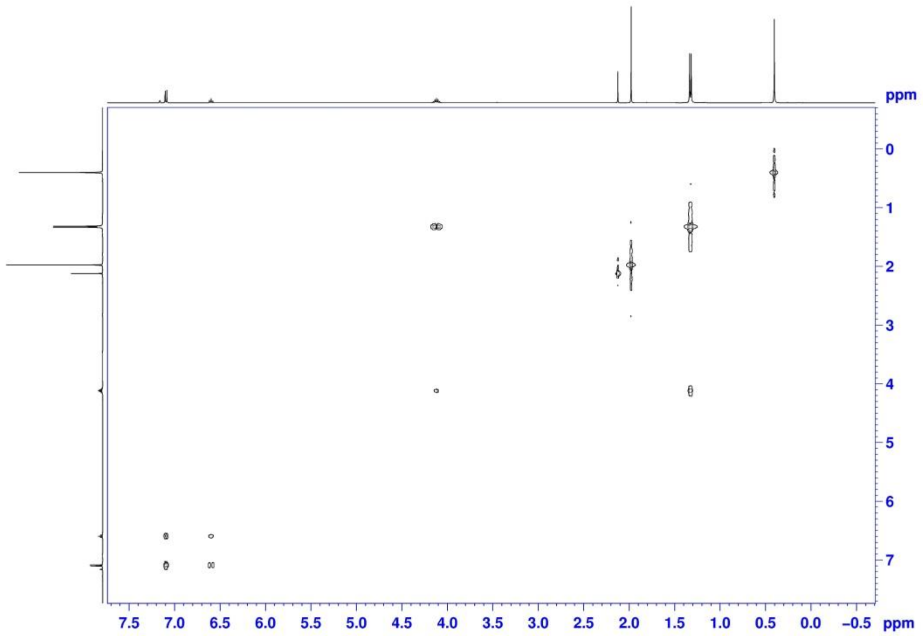

Figure S35. ^{1}H, ^{1}H-COSY NMR spectrum of [{K{N(SiMe$_3$)(Dipp)}}$_2$(TMEDA)]$_\infty$, **8** (400.13 MHz, C_6D_6/[D_8]THF, 25ºC).

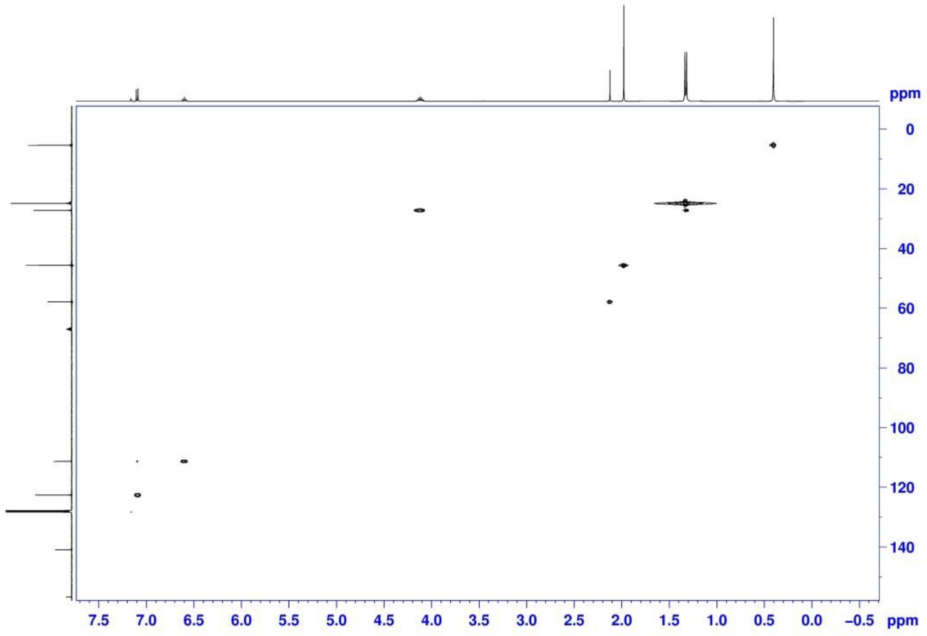

Figure S36. ^{1}H, ^{13}C-HSQC NMR spectrum of [{K{N(SiMe$_3$)(Dipp)}}$_2$(TMEDA)]$_\infty$, **8** (400.13 MHz, C_6D_6/[D_8]THF, 25ºC).

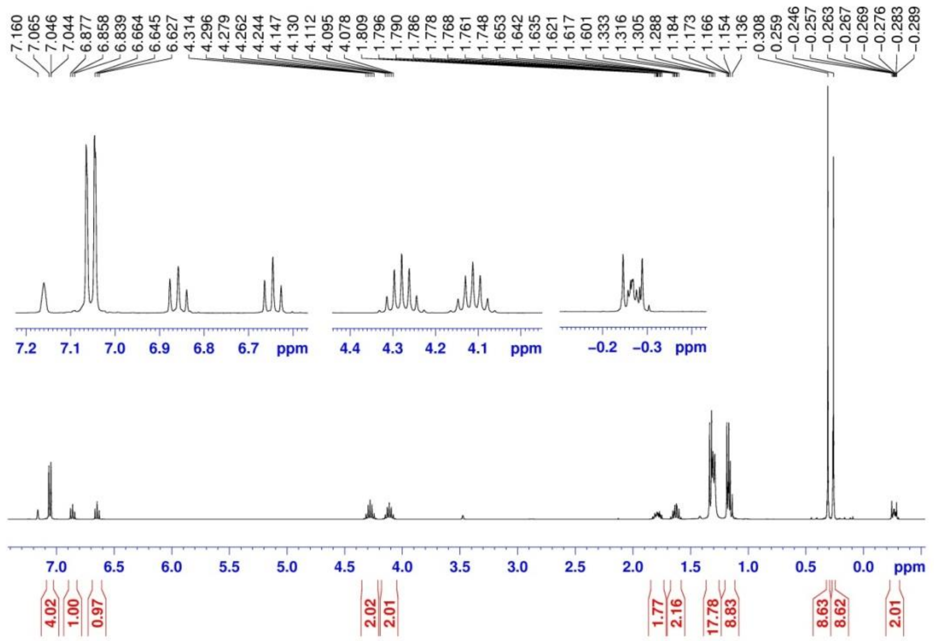

Figure S37. ^{1}H NMR spectrum of [NaMg{N(SiMe$_3$)(Dipp)}$_2$(μ-*n*Bu)]$_\infty$, **9** (400.13 MHz, C_6D_6/[D_8]THF, 25ºC).

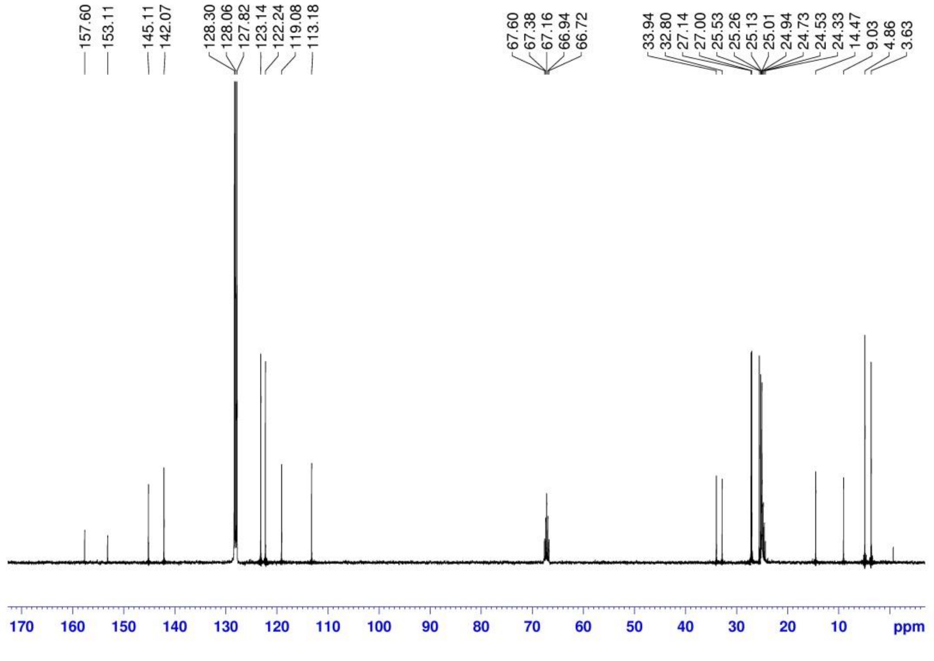

Figure S38. ^{13}C{^{1}H} NMR spectrum of [NaMg{N(SiMe$_3$)(Dipp)}$_2$(μ-*n*Bu)]$_\infty$, **9** (100.6 MHz, C_6D_6/[D_8]THF, 25ºC).

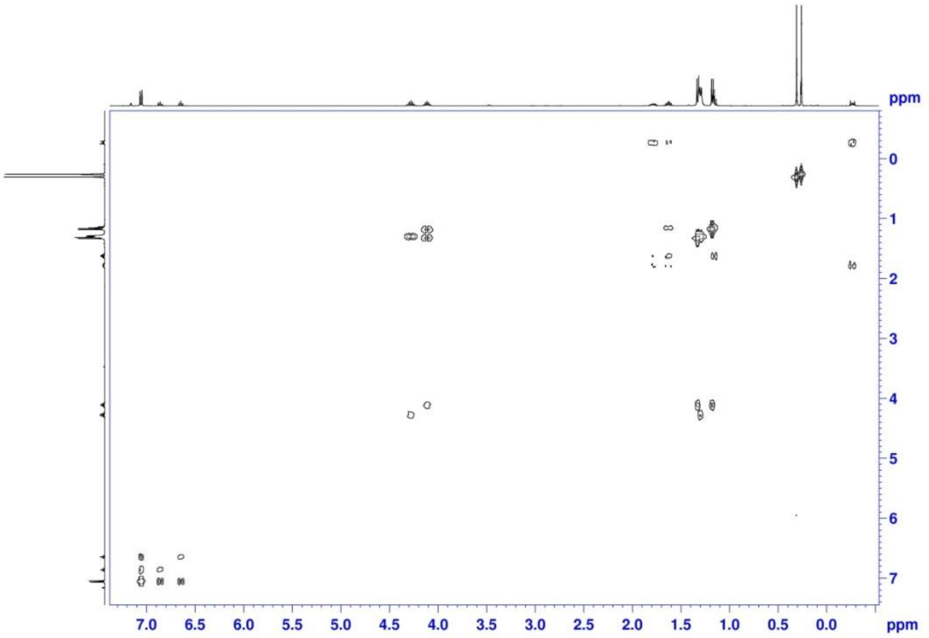

Figure S39. ^{1}H, ^{1}H-COSY NMR spectrum of $[NaMg\{N(SiMe_3)(Dipp)\}_2(\mu\text{-}n\text{Bu})]_\infty$, **9** (400.13 MHz, C_6D_6/$[D_8]$THF, 25ºC).

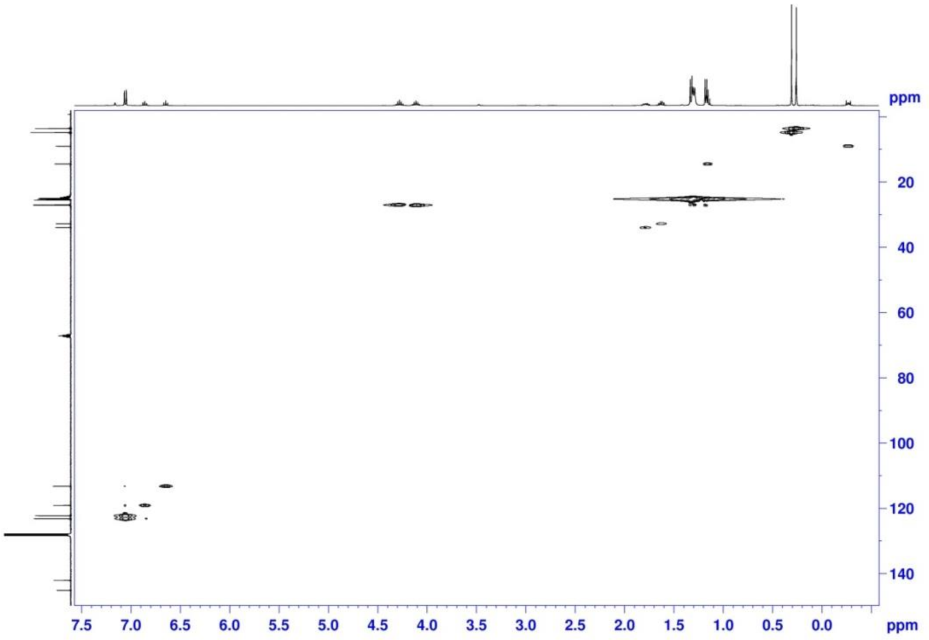

Figure S40. ^{1}H, ^{13}C-HSQC NMR spectrum of $[NaMg\{N(SiMe_3)(Dipp)\}_2(\mu\text{-}n\text{Bu})]_\infty$, **9** (400.13 MHz, C_6D_6/$[D_8]$THF, 25ºC).

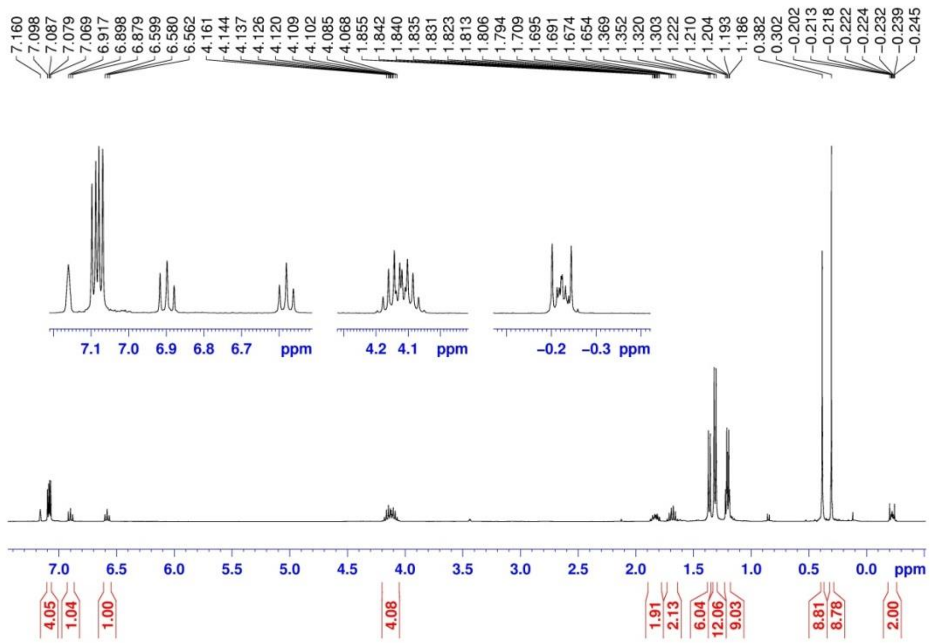

Figure S41. ^{1}H NMR spectrum of [KMg{N(SiMe$_3$)(Dipp)}$_2$(μ-*n*Bu)]$_\infty$, **10** (400.13 MHz, C_6D_6/[D_8]THF, 25ºC).

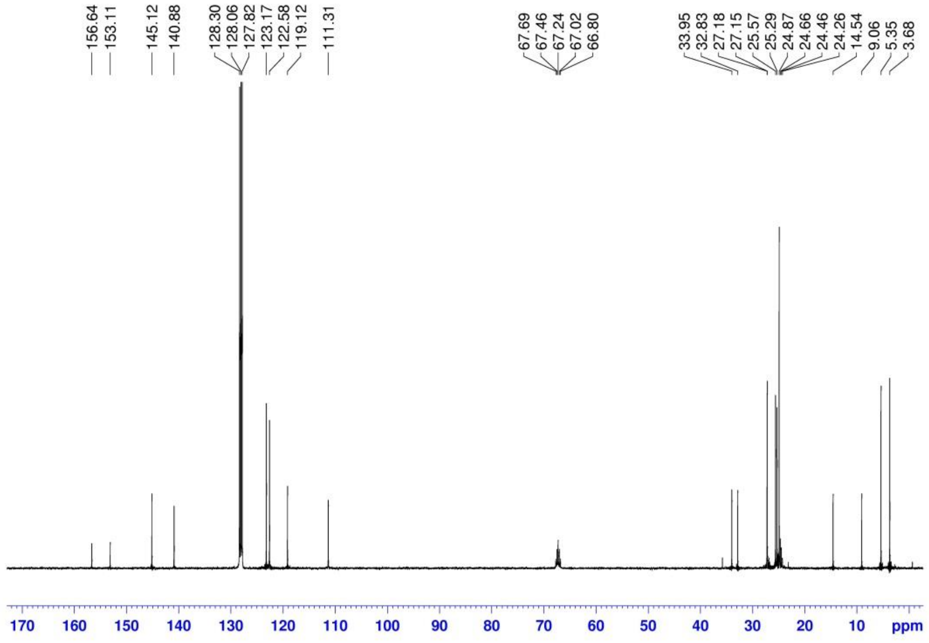

Figure S42. ^{13}C{^{1}H} NMR spectrum of [KMg{N(SiMe$_3$)(Dipp)}$_2$(μ-*n*Bu)]$_\infty$, **10** (100.6 MHz, C_6D_6/[D_8]THF, 25ºC).

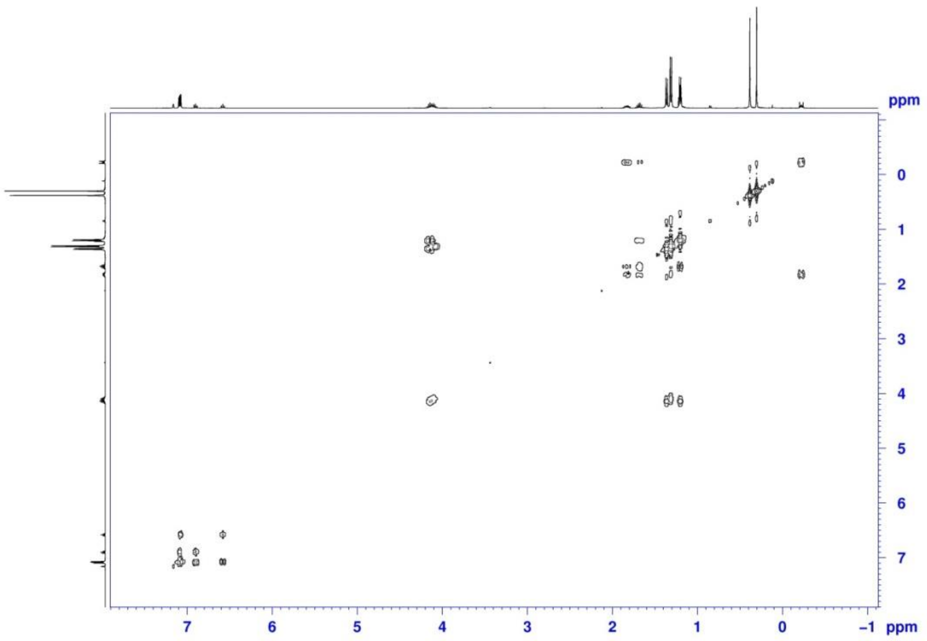

Figure S43. ^{1}H, ^{1}H-COSY NMR spectrum of [KMg{N(SiMe$_3$)(Dipp)}$_2$(μ-*n*Bu)]$_\infty$, **10** (400.13 MHz, C_6D_6/[D$_8$]THF, 25ºC).

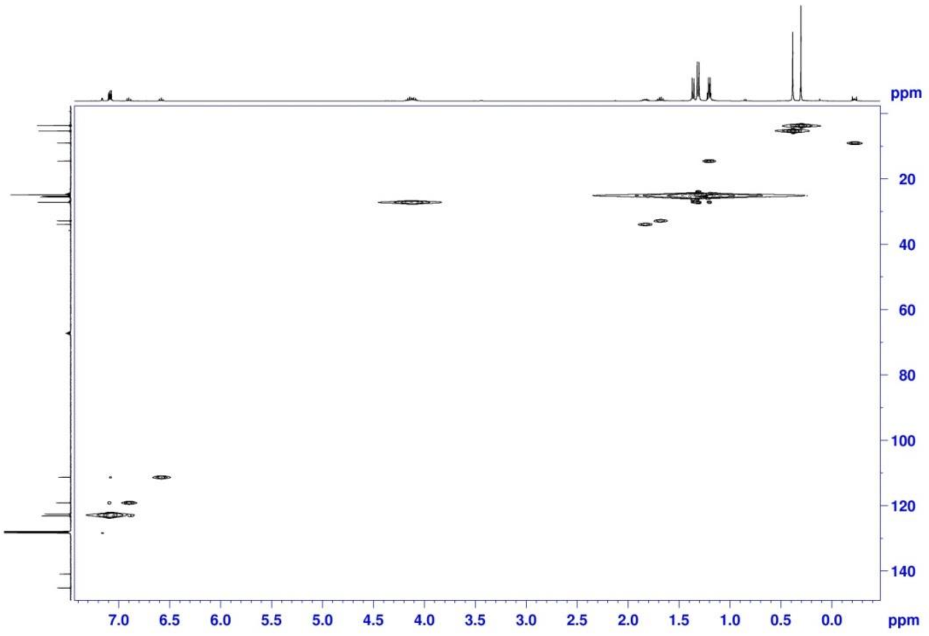

Figure S44. ^{1}H, ^{13}C-HSQC NMR spectrum of [KMg{N(SiMe$_3$)(Dipp)}$_2$(μ-*n*Bu)]$_\infty$, **10** (400.13 MHz, C_6D_6/[D$_8$]THF, 25ºC).

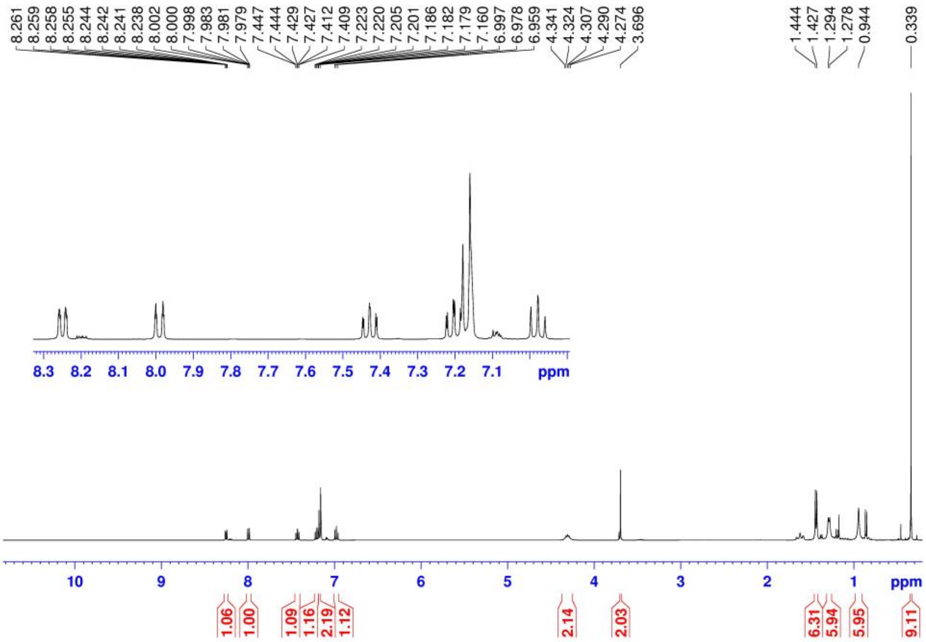

Figure S45. ^{1}H NMR spectrum of [Mg{N(SiMe$_3$)(Dipp)}{2-C$_6$H$_4$-1-(oxazoline(Me)$_2$)}], **11** (400.13 MHz, C$_6$D$_6$/[D$_8$]THF, 25ºC).

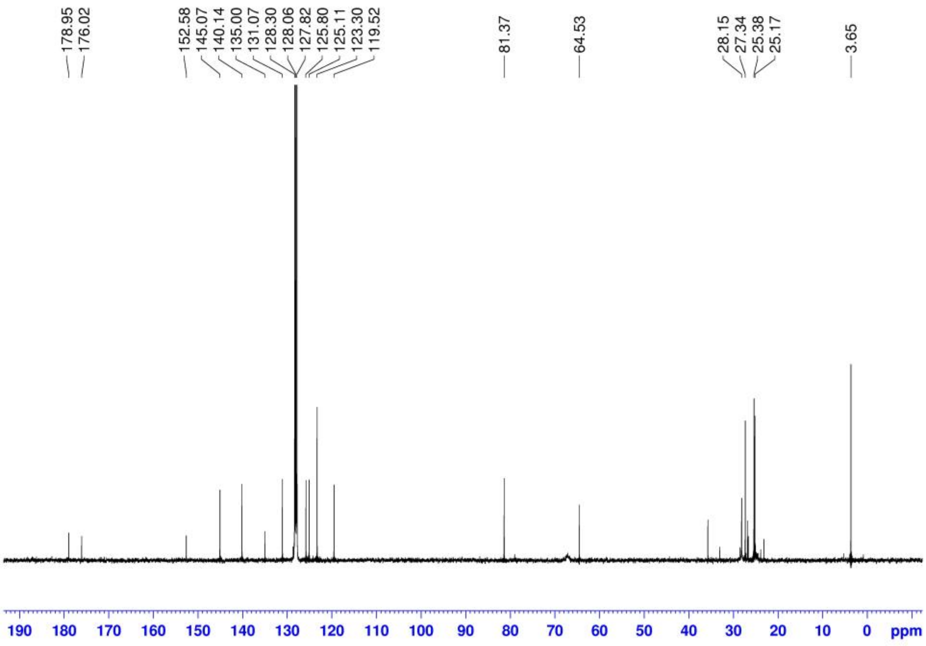

Figure S46. ^{13}C{^{1}H} NMR spectrum of [Mg{N(SiMe$_3$)(Dipp)}{2-C$_6$H$_4$-1-(oxazoline(Me)$_2$)}], **11** (100.6 MHz, C$_6$D$_6$/[D$_8$]THF, 25ºC).

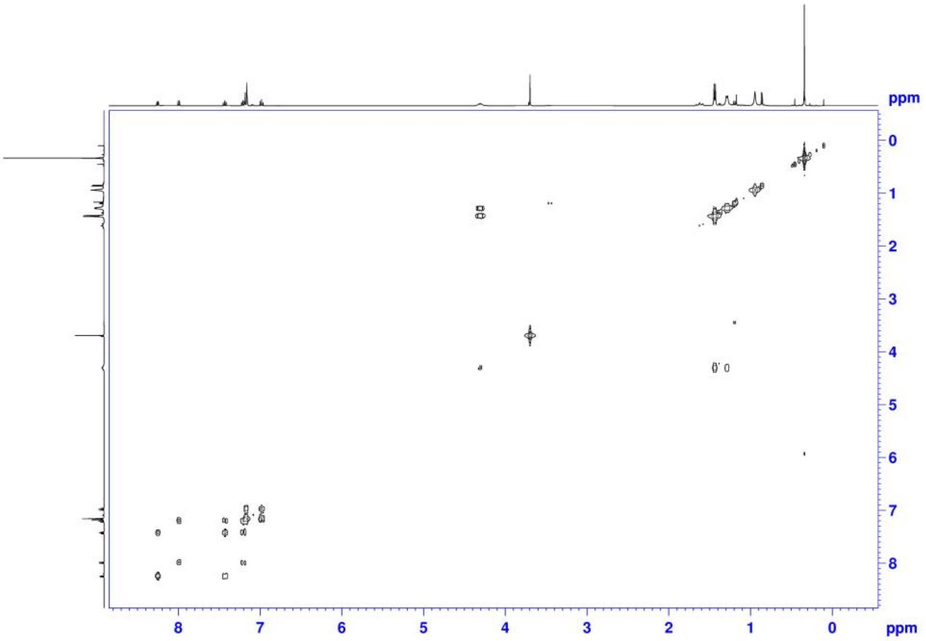

Figure S47. ^{1}H, ^{1}H-COSY NMR spectrum of [Mg{N(SiMe$_3$)(Dipp)}{2-C$_6$H$_4$-1-(oxazoline(Me)$_2$)}], **11** (400.13 MHz, C$_6$D$_6$/[D$_8$]THF, 25ºC).

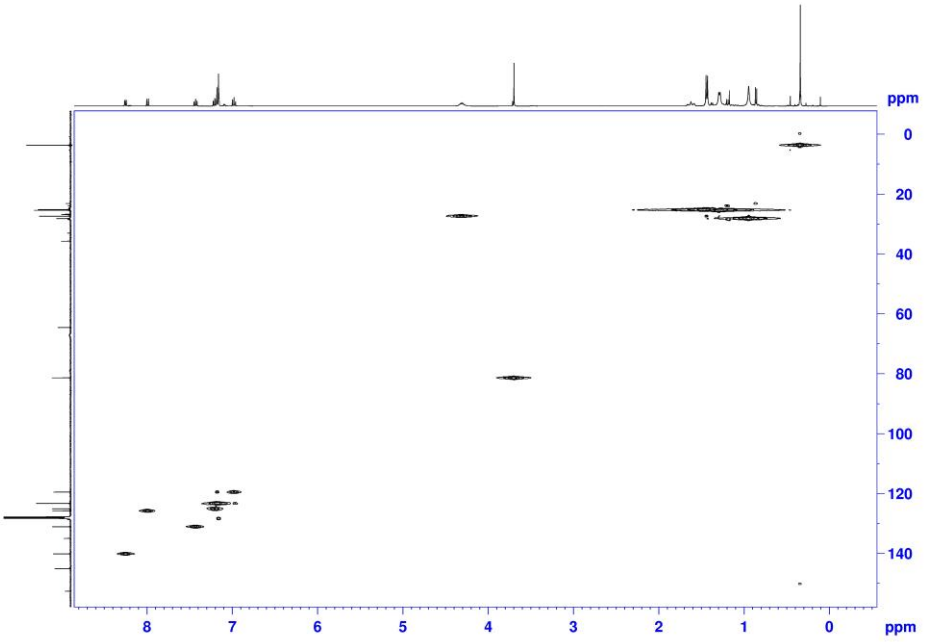

Figure S48. ^{1}H, ^{13}C-HSQC NMR spectrum of [Mg{N(SiMe$_3$)(Dipp)}{2-C$_6$H$_4$-1-(oxazoline(Me)$_2$)}], **11** (400.13 MHz, C$_6$D$_6$/[D$_8$]THF, 25ºC).

CRYSTALLOGRAPHIC DATA

Table S7. Selected crystallographic and refinement data for **1** – **5**.

	**1**	**2**	**3**	**4**	**5**
Formula	C_{15} H_{26} N Na Si[a]	C_{15} H_{26} K N Si	C_{24} H_{49} Li N_4 Si	C_{24} H_{49} N_4 Na Si	C_{24} H_{49} K N_4 Si
Fw	271.45	287.56	428.70	444.75	460.86
Cryst. System	Orthorhombic	Monoclinic	Monoclinic	Monoclinic	Triclinic
Space Group	P n m a	P 2_1/c	P 2_1/n	P 2_1/n	P -1
Wavelength/Å	0.71073	0.71073	0.71073	0.71073	0.71073
a/Å	6.0790(3)	11.6159(4)	11.0999(4)	11.4800(6)	10.7947(8)
b/Å	14.0398(15)	13.5956(4)	16.2073(4)	16.2212(8)	11.5872(8)
c/Å	23.1425(16)	11.6524(3)	15.0567(4)	14.9984(10)	12.2466(8)
α/°	90	90	90	90	92.169(5)
β/°	90	114.035(4)	98.478(3)	98.920(5)	106.769(6)
γ/°	90	90	90	90	101.222(6)
Volume/$Å^3$	1975.2(3)	1680.65(10)	2679.09(14)	2759.2(3)	1431.40(18)
Z	4	4	4	4	2
Temp./K	123(2) K	123(2)	123(2)	123(2)	123(2)
Refls. Collect.	12547	9939	15155	36093	22332
$2\theta_{max}$	59.89	57.98	58.05	60.52	60.49
R_{int}	0.0328	0.0324	0.0313	0.0503	0.0380
Goodness of fit	1.081	1.014	1.032	1.021	1.029
$R[F^2>2\sigma]$, F	0.0501	0.0410	0.0478	0.0486	0.0474
R_w (all data), F^2	0.1314	0.0982	0.1200	0.1176	0.1049

[a] The given formula does not include solvent that was removed from the structure using SQUEEZE. See experimental text for details.

**Table S8**. Selected crystallographic and refinement data for **6** – **10**.					
	**6**	**7**	**8**	**9**	**10**
Formula	$C_{21}H_{42}LiN_3Si$	$C_{21}H_{42}N_3NaSi$	$C_{36}H_{68}K_2N_4Si_2$	$C_{34}H_{61}MgN_2NaSi_2$	$C_{37.50}H_{68}KMgN_2Si_2$
Fw	371.60	387.65	691.32	601.32	666.52
Cryst. System	Orthorhombic	Triclinic	Orthorhombic	Monoclinic	Monoclinic
Space Group	P n a 2_1	P -1	P c a 2_1	C 2/c	C 2/c
Wavelength/Å	0.71073	1.5418	0.71073	0.71073	0.71073
a/Å	15.5243(2)	9.6554(9)	25.7257(6)	21.7784(9)	41.585(3)
b/Å	16.0495(3)	10.1664(7)	9.7313(2)	10.2342(3)	11.8402(4)
c/Å	9.61195(15)	14.0432(11)	33.3128(8)	34.6078(15)	18.3872(13)
α/°	90.0	87.602(6)	90	90	90
β/°	90.0	78.551(7)	90	98.365(4)	116.233(9)
γ/°	90.0	72.108(7)	90	90	90
Volume/$Å^3$	2394.87(7)	1285.45(19)	8339.7(3)	7631.5(5)	8120.9(10)
Z	4	2	8	8	8
Temp./K	123(2)	230(2)	123(2)	123(2)	123(2)
Refls. Collect.	26892	20448	82566	39301	22447
$2\theta_{max}$	59.99	146.53	57.88	57.99	57.99
R_{int}	0.0342	0.0276	0.0389	0.0479	0.0407
Goodness of fit	1.083	1.039	1.087	1.126	1.029
$R[F^2>2\sigma]$, F	0.0441	0.0476	0.0461	0.0594	0.0537
R_w (all data), F^2	0.1029	0.1439	0.0974	0.1322	0.1424

Table S9. Comparison of selected metric data for **1 – 10**.

Complexes		Selected bond lengths [Å]		Selected angles [º]
**Li**	**3**	Li1-N1 2.020(3); Li1-N2 2.314(3); Li1-N3 2.148(3); Li1-N4 2.216(3)		N1-Li1-N2 122.13(13); N1-Li1-N3 125.62(14); N1-Li1-N4 122.30(14); C1-N1-Li1 115.86(13); Si1-N1-Li1 114.96(10)
	**6**	Li1-N1 1.905(4)		Si1-N1-Li1 121.50(15); C1-N1-Li1 108.20(17)
**Na**	**1**	Na1-N1 2.2585(16)	Na1-C4´´ 2.696(2); Na1-C3´´ 2.7514(16)	N1-Na1-C4´´ 130.07(6); N1-Na1-C3´´ 137.70(5); C1-N1-Na1 116.41(11); Si1-N1-Na1 119.77(8)
	**4**	Na1-N1 2.3206(12); Na1-N2 2.4862(13); Na1-N3 2.4188(14); Na1-N4 2.5201(14)	Na1-C1 3.0937(14)	N1-Na1-N2 125.66(5); N1-Na1-N3 125.63(5); N1-Na1-N4 123.87(5); C1-N1-Na1 111.17(8); Si1-N1-Na1 107.98(6)
	**7**	Na1-N1 2.2847(14); Na1-N2 2.4726(17); Na1-N3 2.461(2)	Na1-C14´ 2.869(2); Na1-C1 2.9379(16)	N1-Na1-N3 133.21(7); N1-Na1-N2 130.49(6); C1-N1-Na1 103.88(9); Si1-N1-Na1 124.02(7)
**K**	**2**	K1-N1 2.6755(12)	K1-Centroid´ 2.81	N1-K1-Centroid´ 154.53; C1-N1-K1 93.59(8); Si1-N1-K1 128.62(7)
	**5**	K1-N1 2.7174(13); K1-N2 2.9203(15); K1-N3 2.9306(15); K1-N4 2.8230(15)	K1-C1 2.9806(14); K1-C13´ 3.2272(17); K1-C20 3.2166(2)	N1-K1-N4 99.71(4); N1-K1-N2 127.56(4); N1-K1-N3 157.12(4); N1-K1-C13´ 90.96(4); C1-N1-K1 87.06(8); Si1-N1-K1 139.87(7)
	**8**	K2-N3 2.780(3); K2-N1´ 2.830(3); K2-N2 2.831(3); K2-N4 2.932(3)	K1-Centroid1 2.7943(8); K1-Centroid2 2.7909(8)	Centroid1-K1-Centroid2 165.8; C1´-N1´-K2 112.00(19); Si1´-N1´-K2 110.56(12); N3-K2-N1´ 93.22(9); N3-K2-N2 103.45(9); N1´-K2-N2 148.95(9); N3-K2-N4 66.72(9); N1´-K2-N4 97.86(9); N2-K2-N4 112.70(9); C16-N2-K2 108.7(2); Si2-N2-K2 107.61(13)
**M/Mg**[a]	**9**	Mg1-N1 2.0263(16); Mg1-N2 2.0393(15)	Na1-C16 2.779(2); Na1-C4 2.793(2); Na1-C5 2.913(2); Na2-Centroid 2.5311(1); Mg1-C16 2.1533(19)	Mg1-C16-Na1 126.94(8); N1-Mg1-N2 129.36(7); N1-Mg1-C16 112.50(7); Centroid-Na2-Centroid 180.0
	**10**	Mg1-N1 2.0327(18); Mg1-N2´ 2.0341(17)	K1-C31 3.046(3); K1-Centroid1 2.9162(1); K1-Centroid2 2.9239(1); Mg1-C31 2.150(2)	Centroid1-K1-Centroid2 147.4; Mg1-C31-K1 94.26(9); N1-Mg1-N2´ 133.18(7)

[a] M = Na, K.

REACTIVITY DATA

Table S10. Metallation of **12a** followed by iodination reaction to give **13a**.

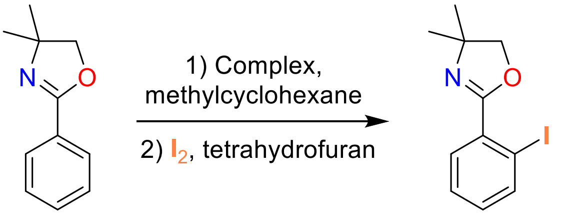

12a **13a**

Entry	Complex	Ratio[b]	*T* (°C)	Yield (%)[c]
1	**1** or **2**	1:1	25	n. r.
2			101	n. r.
3	[Mg][a]	1:1	25	80
4			101	99
5	**9**	1:1	25	10
6			101	69
7		1:2	25	45
8			101	99
9	**10**	1:1	25	11
10			101	88
11		1:2	25	15
12			101	99

[a] [Mg] corresponds to the complex [Mg{N(SiMe$_3$)(Dipp)}(*n*Bu)] made *in situ*. [b] Stoichiometric ratio between the substrate and the complex respectively. [c] Yield was calculated from ^{1}H NMR spectra using 1,10-phenanthroline as internal standard. n.r. stands for no reaction.

Table S11. Metallation of **12b** followed by iodination reaction to give **13b**.

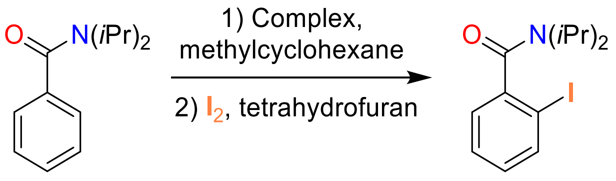

12b **13b**

Entry	Complex	Ratio[b]	*T* (°C)	Yield (%)[c]
1	**1** or **2**	1:1	25	n. r.
2			101	n. r.
3	[Mg][a]	1:1	25	n. r.
4			101	42
5	**9**	1:1	25	10
6			101	33
7		1:2	25	8
8			101	31
9	**10**	1:1	25	12
10			101	56
11		1:2	25	14
12			101	55

[a] [Mg] corresponds to the complex [Mg{N(SiMe$_3$)(Dipp)}(*n*Bu)] made *in situ*. [b] Stoichiometric ratio between the substrate and the complex respectively. [c] Yield was calculated from ^{1}H NMR spectra using 1,10-phenanthroline as internal standard. n.r. stands for no reaction.

REFERENCES

[1] G. M. Sheldrick, *Acta Cryst. A* **2008**, *64*, 112-122.

[2] A. L. Spek, *Acta Cryst. D* **2009**, *65*, 148-155.

[3] a) R. Neufeld, D. Stalke, *Chem. Sci.* **2015**, *6*, 3354-3364; b) S. Bachmann, R. Neufeld, M. Dzemski, D. Stalke, *Chem. Eur. J.* **2016**, doi: 10.1002/chem.201601145.

Synthesis of [Na{N(SiMe$_3$)(Dipp)}]$_\infty$, 1

In a Schlenk tube, freshly prepared *n*-butylsodium (88.1 mg, 1.1 mmol) was suspended in methylcyclohexane (5 mL) and then 2,6-diisopropyl-*N*-(trimethylsilyl)aniline (249.5 mg, 1 mmol) was added. The reaction mixture was stirred for 2h at room temperature to give a beige suspension. Toluene (3-4 mL) was added and the reaction was heated until a pale yellow solution was obtained. Placed in a Dewar flask of hot water, the solution afforded crystalline complex **1** suitable for an X-ray diffraction study (210 mg, 0.77 mmol, 77%). ^{1}H NMR (400.13 MHz, C_6D_6/[D$_8$]THF, 25°C): δ=7.15 (d, *J*(H,H)=7.4 Hz, 2H; *m*-C*H*-Ar), 6.76 (t, *J*(H,H)=7.4 Hz, 1H; *p*-C*H*-Ar), 4.35 (sept, *J*(H,H)=7.0 Hz, 2H; -C*H*(CH$_3$)$_2$), 1.37 (d, *J*(H,H)=6.9 Hz, 12H; -CH(C*H*$_3$)$_2$), 0.41 ppm (s, 9H; -Si(C*H*$_3$)$_3$). ^{13}C{^{1}H} NMR (100.6 MHz, C_6D_6/[D$_8$]THF, 25°C): δ=157.5 (N-*C*$_q$-Ar), 142.1 (*o*-*C*$_q$-Ar), 122.3 (*m*-*C*H-Ar), 113.3 (*p*-*C*H-Ar), 27.0 (-*C*H(CH$_3$)$_2$), 25.0 (-CH(*C*H$_3$)$_2$), 4.9 ppm (-Si(*C*H$_3$)$_3$). Elemental analysis calcd. (%) for $C_{15}H_{26}NNaSi$: C 66.37, H 9.65, N 5.16; found: C 65.93, H 9.64, N 5.23.

Synthesis of [K{N(SiMe$_3$)(Dipp)}]$_\infty$, 2

Prepared in an analogous manner to **1** using KCH$_2$SiMe$_3$ (138.9 mg, 1.1 mmol) as metallating reagent (207 mg, 0.72 mmol, 72%). ^{1}H NMR (400.13 MHz, C_6D_6/[D$_8$]THF, 25°C): δ=7.06 (d, *J*(H,H)=7.4 Hz, 2H; *m*-C*H*-Ar), 6.55 (t, *J*(H,H)=7.3 Hz, 1H; *p*-C*H*-Ar), 4.08 (sept, *J*(H,H)=6.9 Hz, 2H; -C*H*(CH$_3$)$_2$), 1.30 (d, *J*(H,H)=6.9 Hz, 12H; -CH(C*H*$_3$)$_2$), 0.38 ppm (s, 9H; -Si(C*H*$_3$)$_3$). ^{13}C{^{1}H} NMR (100.6 MHz, C_6D_6/[D$_8$]THF, 25°C): δ=156.8 (N-*C*$_q$-Ar), 141.0 (*o*-*C*$_q$-Ar), 122.6 (*m*-*C*H-Ar), 111.1 (*p*-*C*H-Ar), 27.1 (-*C*H(CH$_3$)$_2$), 24.8 (-CH(*C*H$_3$)$_2$), 5.3 ppm (-Si(*C*H$_3$)$_3$). Elemental analysis calcd. (%) for $C_{15}H_{26}NKSi$: C 62.65, H 9.11, N 4.87; found: C 62.17, H 9.07, N 4.80.

Synthesis of [Li{N(SiMe$_3$)(Dipp)}(PMDETA)], 3

Commercial *n*-butyllithium (0.69 mL, 1.6M solution in *n*-hexane, 1.1 mmol) was added via syringe to a Schlenk tube containing a solution of 2,6-diisopropyl-*N*-(trimethylsilyl)aniline (249.5 mg, 1 mmol) in *n*-hexane (5 mL) under argon atmosphere. The resulting suspension was stirred for 15 minutes to give the white solid Li[N(SiMe$_3$)(Dipp)]. PMDETA (0.23 mL, 1.1 mmol) was introduced and the reaction was stirred for 1h at room temperature giving a yellow suspension. Toluene (0.5-1 mL) was added and the reaction was heated until a solution was obtained.

Placed in a Dewar flask containing hot water to give crystalline complex **3** suitable for an X-ray diffraction study. The crystals were collected, washed with *n*-hexane (3x1 mL) and dried under vacuum (205 mg, 0.48 mmol, 48%). Full conversion by ^{1}H NMR evidence. ^{1}H NMR (400.13 MHz, C_6D_6, 25°C): δ=7.20 (d, *J*(H,H)=7.2 Hz, 2H; *m*-C*H*-Ar), 6.90 (t, *J*(H,H)=7.6 Hz, 1H; *p*-C*H*-Ar), 4.16 (sept, *J*(H,H)=6.9 Hz, 2H; -C*H*(CH_3)$_2$), 1.84 (s, 17H; -CH_2- + CH_3-PMDETA), 1.72 (m, 4H; -CH_2-PMDETA), 1.63 (br s, 2H; -CH_2-PMDETA), 1.47 (br d, *J*(H,H)=6.0 Hz, 6H; -CH(CH_3)$_2$), 1.32 (br d, *J*(H,H)=6.4 Hz, 6H; -CH(CH_3)$_2$), 0.39 ppm (s, 9H; -Si(CH_3)$_3$). ^{7}Li NMR (155.5 MHz, C_6D_6, 25°C): δ=0.07 ppm. ^{13}C{^{1}H} NMR (100.6 MHz, C_6D_6, 25°C): δ=157.8 (N-C_q-Ar), 143.1 (*o*-C_q-Ar), 122.7 (*m*-*C*H-Ar), 115.6 (*p*-*C*H-Ar), 57.6 (-*C*H_2-PMDETA), 53.9 (-*C*H_2-PMDETA), 46.0 (terminal *C*H_3-PMDETA), 44.9 (central *C*H_3-PMDETA), 26.9 (-*C*H(CH_3)$_2$), 26.4 (-CH(*C*H_3)$_2$), 25.2 (-CH(*C*H_3)$_2$), 5.4 ppm (-Si(*C*H_3)$_3$). Elemental analysis calcd. (%) for $C_{24}H_{49}LiN_4Si$: C 67.24, H 11.52, N 13.07; found: C 67.58, H 12.01, N 13.13.

Synthesis of [Na{N(SiMe$_3$)(Dipp)}(PMDETA)], 4

Method A: 1 mmol of **1** was prepared in *n*-hexane (5 mL). PMDETA (0.23 mL, 1.1 mmol) was added and the reaction was stirred for 1h at room temperature giving a beige suspension. Toluene (0.5-1 mL) was added and the reaction was heated slightly until a solution was realised. This was placed in a refrigerator operating at 8°C to give crystalline complex **4** suitable for an X-ray diffraction study. The crystals were collected, washed with *n*-hexane (3x1 mL) and dried under vacuum (256 mg, 0.58 mmol, 58%).

Method B: 1 mmol of **3** was prepared in *n*-hexane. Commercial unrefined sodium *tert*-butoxide (105.7 mg, 1.1 mmol) was added to the reaction mixture under argon atmosphere. The resulting suspension mixture was stirred for 2h then heated gently until a yellow solution resulted. Refrigeration of this solution at 8°C produced after 12h crystals of **4**. These were filtered, washed with *n*-hexane (3x1 mL) and dried under vacuum (233 mg, 0.52 mmol, 52%). ^{1}H NMR (400.13 MHz, C_6D_6, 25°C): δ=7.19 (d, *J*(H,H)=7.2 Hz, 2H; *m*-C*H*-Ar), 6.81 (t, *J*(H,H)=7.6 Hz, 1H; *p*-C*H*-Ar), 4.22 (sept, *J*(H,H)=7.0 Hz, 2H; -C*H*(CH_3)$_2$), 1.85 (s, 3H; CH_3-PMDETA), 1.70 (s, 12H; CH_3-PMDETA), 1.68-1.48 (m, 8H; -CH_2-PMDETA), 1.40 (br s, 12H; -CH(CH_3)$_2$), 0.45 ppm (s, 9H; -Si(CH_3)$_3$). ^{13}C{^{1}H} NMR (100.6 MHz, C_6D_6, 25°C): δ=157.8 (N-C_q-Ar), 141.5 (*o*-C_q-Ar), 122.6 (*m*-*C*H-Ar), 113.4 (*p*-*C*H-Ar), 57.2 (-*C*H_2-PMDETA), 53.6 (-*C*H_2-PMDETA), 45.3 (terminal *C*H_3-PMDETA), 43.9 (central *C*H_3-PMDETA), 27.3 (-

CH(CH_3)$_2$), 25.1 (br-CH(CH_3)$_2$), 5.3 ppm (-Si(CH_3)$_3$). Elemental analysis calcd. (%) for $C_{24}H_{49}N_4NaSi$: C 64.81, H 11.10, N 12.60; found: C 64.71, H 11.40, N 12.57.

Synthesis of [K{N(SiMe$_3$)(Dipp)}(PMDETA)]$_2$, 5

Method A: 1 mmol of **2** was prepared in methylcyclohexane (5 mL) and the reaction mixture was stirred for 0.5h. PMDETA (0.23 mL, 1.1 mmol) was added and the reaction was stirred for 1h at room temperature. Warmed slightly until homogeneous, the solution was placed in the refrigerator at 8°C to give crystalline complex **5** suitable for an X-ray diffraction study. The crystals were collected, washed with *n*-hexane (3x1 mL) and dried under vacuum (261 mg, 0.56 mmol, 56%).

Method B: 1 mmol of **3** was prepared in methylcyclohexane. Commercial unrefined potassium *tert*-butoxide (123.4 mg, 1.1 mmol) was added to the reaction mixture under argon atmosphere and was stirred for 2h. The reaction mixture was heated slightly during the addition of methylcyclohexane (2 mL) until a yellow solution was obtained and immediately placed in the refrigerator at 8°C. Crystals of **5** were obtained. They were filtered, washed with *n*-hexane (3x1 mL) and dried under vacuum (232 mg, 0.50 mmol, 50%). ^{1}H NMR (400.13 MHz, C_6D_6, 25°C): δ=7.17 (br d, 2H; *m*-C*H*-Ar), 6.69 (v br t, *J*(H,H)=7.0 Hz, 1H; *p*-C*H*-Ar), 4.16 (br m, 2H; -C*H*(CH_3)$_2$), 1.74 (s, 12H; C*H*$_3$-PMDETA), 1.69 (s, 8H; -C*H*$_2$-PMDETA), 1.55 (s, 3H; C*H*$_3$-PMDETA), 1.39 (d, *J*(H,H)=6.8 Hz, 12H; -CH(C*H*$_3$)$_2$), 0.53 ppm (br s, 9H; -Si(C*H*$_3$)$_3$). ^{13}C{^{1}H} NMR (100.6 MHz, C_6D_6, 25°C): δ=157.0 (N-*C*$_q$-Ar), 140.6 (*o*-*C*$_q$-Ar), 122.8 (*m*-*C*H-Ar), 111.3 (*p*-*C*H-Ar), 56.8 (-*C*H$_2$-PMDETA), 55.1 (-*C*H$_2$-PMDETA), 44.9 (terminal *C*H$_3$-PMDETA), 40.9 (central *C*H$_3$-PMDETA), 27.4 (-*C*H(CH_3)$_2$), 24.8 (br-CH(*C*H$_3$)$_2$), 5.7 ppm (-Si(*C*H$_3$)$_3$). Elemental analysis calcd. (%) for $C_{24}H_{49}KN_4Si$: C 62.55, H 10.72, N 12.16; found: C 63.16, H 11.02, N 13.05.

Synthesis of [Li{N(SiMe$_3$)(Dipp)}(TMEDA)], 6

Prepared in an analogous manner to **3** using TMEDA (0.16 mL, 1.1 mmol). Stored in the refrigerator at 8°C the solution deposited crystalline complex **6** suitable for an X-ray diffraction study. The crystals were collected, washed with *n*-hexane (3x1 mL) and dried under vacuum (295 mg, 0.79 mmol, 79%). Full conversion was confirmed by ^{1}H-NMR spectroscopy. ^{1}H NMR (400.13 MHz, C_6D_6, 25°C): δ=7.20 (d, *J*(H,H)=7.6 Hz, 2H; *m*-C*H*-Ar), 6.90 (t, *J*(H,H)=7.6 Hz, 1H; *p*-C*H*-Ar), 4.04 (sept, *J*(H,H)=6.8 Hz, 2H; -C*H*(CH_3)$_2$), 1.61 (s, 12H; C*H*$_3$-TMEDA), 1.44 (s, 4H; -C*H*$_2$-TMEDA), 1.44-1.34

(br s, 12H; -CH(CH_3)$_2$), 0.42 ppm (s, 9H; -Si(CH_3)$_3$). ^{7}Li NMR (155.5 MHz, C_6D_6, 25°C): δ=0.54 ppm. ^{13}C{^{1}H} NMR (100.6 MHz, C_6D_6, 25°C): δ=156.0 (N-C_q-Ar), 142.9 (*o*-C_q-Ar), 122.5 (*m*-*C*H-Ar), 115.7 (*p*-*C*H-Ar), 56.1 (-*C*H_2-TMEDA), 44.7 (*C*H_3-TMEDA), 27.6 (-*C*H(CH$_3$)$_2$), 25.8 (-CH(*C*H$_3$)$_2$), 24.5 (-CH(*C*H$_3$)$_2$), 4.9 ppm (-Si(*C*H$_3$)$_3$). Elemental analysis calcd. (%) for $C_{21}H_{42}LiN_3Si$: C 67.87, H 11.39, N 11.31; found: C 67.89, H 11.43, N 11.56.

Synthesis of [Na{N(SiMe$_3$)(Dipp)}(TMEDA)]$_2$, 7

1 mmol of **1** was prepared in *n*-hexane (5 mL). TMEDA (0.16 mL, 1.1 mmol) was added and the reaction was stirred for 1h at room temperature. Toluene ($<$0.5 mL) was added and the reaction was heated slightly until a solution was obtained. The Schlenk tube was kept at room temperature. Crystals of **7** were obtained which were suitable for an X-ray diffraction study. They were filtered, washed with *n*-hexane (3x1 mL) and dried under vacuum (172 mg, 0.45 mmol, 45%). ^{1}H NMR (400.13 MHz, C_6D_6, 25°C): δ=7.23 (d, *J*(H,H)=7.6 Hz, 2H; *m*-C*H*-Ar), 6.85 (t, *J*(H,H)=7.4 Hz, 1H; *p*-C*H*-Ar), 4.26 (sept, *J*(H,H)=6.9 Hz, 2H; -C*H*(CH$_3$)$_2$), 1.57 (s, 12H; CH_3-TMEDA), 1.49 (s, 4H; -CH_2-TMEDA), 1.39 (d, *J*(H,H)=7.2 Hz, 12H; -CH(CH_3)$_2$), 0.44 ppm (s, 9H; -Si(CH_3)$_3$). ^{13}C{^{1}H} NMR (100.6 MHz, C_6D_6, 25°C): δ=156.7 (N-C_q-Ar), 141.7 (*o*-C_q-Ar), 122.7 (*m*-*C*H-Ar), 114.0 (*p*-*C*H-Ar), 56.5 (-*C*H$_2$-TMEDA), 45.1 (*C*H$_3$-TMEDA), 27.3 (-*C*H(CH$_3$)$_2$), 25.0 (-CH(*C*H$_3$)$_2$), 5.1 ppm (-Si(*C*H$_3$)$_3$). Elemental analysis calcd. (%) for $C_{21}H_{42}N_3NaSi$: C 65.06, H 10.92, N 10.84; found: C 64.93, H 11.33, N, 10.85.

Synthesis of [{K{N(SiMe$_3$)(Dipp)}}$_2$(TMEDA)]$_\infty$, 8

1 mmol of **2** was prepared in *n*-hexane (5 mL). TMEDA (0.16 mL, 1.1 mmol) was added and the reaction was stirred for 1h at room temperature. Toluene was added and the reaction was heated slightly until a solution was obtained. The solution was cooled down slowly to room temperature giving crystalline complex **8** suitable for an X-ray diffraction study. The crystals were collected, washed with *n*-hexane (3x1 mL) and dried under vacuum (350 mg, 0.50 mmol, 50%). ^{1}H NMR (400.13 MHz, C_6D_6/[D_8]THF, 25°C): δ=7.09 (d, *J*(H,H)=7.6 Hz, 4H; *m*-C*H*-Ar), 6.59 (t, *J*(H,H)=7.4 Hz, 2H; *p*-C*H*-Ar), 4.12 (sept, *J*(H,H)=6.9 Hz, 4H; -C*H*(CH$_3$)$_2$), 2.12 (s, 4H; -CH_2-TMEDA), 1.97 (s, 12H; CH_3-TMEDA), 1.32 (d, *J*(H,H)=7.2 Hz, 24H; -CH(CH_3)$_2$), 0.40 ppm (s, 18H; -Si(CH_3)$_3$). ^{13}C{^{1}H} NMR (100.6 MHz, C_6D_6/[D_8]THF, 25°C): δ=156.7 (N-C_q-Ar), 140.8 (*o*-C_q-Ar), 122.6 (*m*-*C*H-Ar), 111.3 (*p*-*C*H-Ar), 57.8 (-*C*H$_2$-TMEDA), 45.6

(CH_3-TMEDA), 27.2 (-*C*H(CH_3)$_2$), 24.8 (-CH(*C*H_3)$_2$), 5.3 ppm (-Si(*C*H_3)$_3$). Elemental analysis calcd. (%) for $C_{36}H_{68}K_2N_4Si_2$: C 62.55, H 9.91, N 8.10; found: C 62.73, H 9.83, N, 8.47.

Synthesis of [NaMg{N(SiMe$_3$)(Dipp)}$_2$(*μ*-*n*Bu)]$_\infty$, 9

In a Schlenk tube, freshly prepared *n*-butylsodium (88.1 mg, 1.1 mmol) was suspended in methylcyclohexane (5 mL) and then 2,6-diisopropyl-*N*-(trimethylsilyl)aniline (499.0 mg, 2 mmol) was added. The resulting beige suspension was stirred for 1h and then commercial *n*Bu_2Mg (1.1 mL, 1M solution in *n*-heptane, 1.1 mmol) was added via syringe. The reaction mixture was stirred for 1h. Removing all the solvent in vacuo, a mixture of *n*-hexane/toluene was added and it was heated until a yellow solution was obtained. Placed in a Dewar flask filled with hot water to give crystalline complex **9** suitable for an X-ray diffraction study. These were filtered, washed with *n*-hexane (3x4 mL) and dried under vacuum (460 mg, 0.76 mmol, 76%). In addition, reaction studies were carried out using 3:1 amido:butyl stoichiometric ratio which again afforded complex **9**. ^{1}H NMR (400.13 MHz, C_6D_6/[D_8]THF, 25°C): δ=7.05 (d, *J*(H,H)=7.6 Hz, 4H; *m*-C*H*-Ar), 6.85 (t, *J*(H,H)=7.6 Hz, 1H; *p*-C*H*-Ar), 6.64 (t, *J*(H,H)=7.4 Hz, 1H; *p*-C*H*-Ar), 4.27 (sept, *J*(H,H)=7.0 Hz, 2H; -C*H*(CH_3)$_2$), 4.11 (sept, *J*(H,H)=6.9 Hz, 2H; -C*H*(CH_3)$_2$), 1.78 (m, 2H; -CH_2-*n*Bu), 1.63 (m, 2H; -CH_2-*n*Bu), 1.32 (d, *J*(H,H)=6.8 Hz, 6H; -CH(CH_3)$_2$), 1.29 (br d, *J*(H,H)=6.8 Hz, 12H; -CH(CH_3)$_2$), 1.17 (d, *J*(H,H)=7.2 Hz, 6H; -CH(CH_3)$_2$), 1.15 (t, *J*(H,H)=7.4 Hz, 3H; CH_3-*n*Bu), 0.30 (s, 9H; -Si(CH_3)$_3$), 0.25 (s, 9H; -Si(CH_3)$_3$), -0.26 ppm (m, 2H; -CH_2-*n*Bu). ^{13}C{^{1}H} NMR (100.6 MHz, C_6D_6/[D_8]THF, 25°C): δ=157.6 (N-C_q-Ar), 153.1 (N-C_q-Ar), 145.1 (*o*-C_q-Ar), 142.0 (*o*-C_q-Ar), 123.1 (*m*-*C*H-Ar), 122.2 (*m*-*C*H-Ar), 119.0 (*p*-*C*H-Ar), 113.1 (*p*-*C*H-Ar), 33.9 (-*C*H_2-*n*Bu), 32.8 (-*C*H_2-*n*Bu), 27.1 (-*C*H(CH_3)$_2$), 27.0 (-*C*H(CH_3)$_2$), 25.5 (-CH(*C*H_3)$_2$), 25.2 (-CH(*C*H_3)$_2$), 25.0 (br, -CH(*C*H_3)$_2$), 14.4 (*C*H_3-*n*Bu), 9.3 (-*C*H_2-*n*Bu), 4.8 (-Si(*C*H_3)$_3$), 3.6 ppm (-Si(*C*H_3)$_3$). Elemental analysis calcd. (%) for $C_{34}H_{60}MgN_2NaSi_2$: C 68.02, H 10.07, N 4.67; found: C 68.07, H 10.03, N, 4.77.

Synthesis of [KMg{N(SiMe$_3$)(Dipp)}$_2$(*μ*-*n*Bu)]$_\infty$, 10

Prepared in an analogous manner to **9** using KCH_2SiMe_3 (138.9 mg, 1.1 mmol) as metallating reagent (390 mg, 0.63 mmol, 63%). In addition, reaction studies were carried out using 3:1 amido:butyl stoichiometric ratio which again produced complex

10. ^{1}H NMR (400.13 MHz, C_6D_6/[D_8]THF, 25°C): δ=7.08 (d, *J*(H,H)=7.6 Hz, 2H; *m*-C*H*-Ar), 7.07 (d, *J*(H,H)=7.2 Hz, 2H; *m*-C*H*-Ar), 6.89 (t, *J*(H,H)=7.6 Hz, 1H; *p*-C*H*-Ar), 6.58 (t, *J*(H,H)=7.4 Hz, 1H; *p*-C*H*-Ar), 4.14 (sept, *J*(H,H)=6.9 Hz, 2H; -C*H*(CH_3)$_2$), 4.10 (sept, *J*(H,H)=6.9 Hz, 2H; -C*H*(CH_3)$_2$), 1.83 (m, 2H; -CH_2-*n*Bu), 1.67 (m, 2H; -CH_2-*n*Bu), 1.36 (d, *J*(H,H)=6.8 Hz, 6H; -CH(CH_3)$_2$), 1.31 (d, *J*(H,H)=6.8 Hz, 12H; -CH(CH_3)$_2$), 1.20 (d, *J*(H,H)=6.8 Hz, 6H; -CH(CH_3)$_2$), 1.20 (t, *J*(H,H)=7.2 Hz, 3H; CH_3-*n*Bu), 0.38 (s, 9H; -Si(CH_3)$_3$), 0.30 (s, 9H; -Si(CH_3)$_3$), -0.22 ppm (m, 2H; -CH_2-*n*Bu). ^{13}C{^{1}H} NMR (100.6 MHz, C_6D_6/[D_8]THF, 25°C): δ=156.6 (N-C_q-Ar), 153.1 (N-C_q-Ar), 145.1 (*o*-C_q-Ar), 140.8 (*o*-C_q-Ar), 123.1 (*m*-*C*H-Ar), 122.5 (*m*-*C*H-Ar), 119.1 (*p*-*C*H-Ar), 111.3 (*p*-*C*H-Ar), 33.9 (-CH_2-*n*Bu), 32.8 (-CH_2-*n*Bu), 27.2 (-*C*H(CH_3)$_2$), 27.1 (-*C*H(CH_3)$_2$), 25.5 (-CH(CH_3)$_2$), 25.3 (-CH(CH_3)$_2$), 24.8 (-CH(CH_3)$_2$), 14.5 (CH_3-*n*Bu), 9.0 (-CH_2-*n*Bu), 5.3 (-Si(CH_3)$_3$), 3.6 ppm (-Si(CH_3)$_3$). Elemental analysis calcd. (%) for $C_{34}H_{60}KMgN_2Si_2$: C 66.25, H 9.81, N 4.54; found: C 65.97, H 10.04, N 4.95.
